# Radiomics score: a potential prognostic imaging feature for postoperative survival of solitary HCC patients

**DOI:** 10.1186/s12885-018-5024-z

**Published:** 2018-11-21

**Authors:** Bo-Hao Zheng, Long-Zi Liu, Zhi-Zhi Zhang, Jie-Yi Shi, Liang-Qing Dong, Ling-Yu Tian, Zhen-bin Ding, Yuan Ji, Sheng-Xiang Rao, Jian Zhou, Jia Fan, Xiao-Ying Wang, Qiang Gao

**Affiliations:** 1 Department of Liver Surgery and Transplantation, Liver Cancer Institute, Zhongshan Hospital and Key Laboratory of Carcinogenesis and Cancer Invasion, Fudan University, 180 Fenglin Road, Shanghai, 200032 China; 2 Institute of Biomedical Sciences, Fudan University, Shanghai, 200032 China; 3 Department of Pathology, Zhongshan Hospital, Fudan University, Shanghai, 200032 China; 4 Department of Radiology, Zhongshan Hospital, Fudan University, Shanghai, 200032 China; 5 Department of Hematology, Shanghai Jiao Tong University School of Medicine Affiliated Tongren Hospital, Shanghai, China; 6 State Key Laboratory of Genetic Engineering, Fudan University, Shanghai, People’s Republic of China

**Keywords:** Hepatocellular carcinoma, Prognosis, Multidetector computed tomography, Nomogram

## Abstract

**Background:**

Radiomics is an emerging field in oncological research. In this study, we aimed at developing a radiomics score (rad-score) to estimate postoperative recurrence and survival in patients with solitary hepatocellular carcinoma (HCC).

**Methods:**

A total of 319 solitary HCC patients (training cohort: *n* = 212; validation cohort: *n* = 107) were enrolled. Radiomics features were extracted from the artery phase of preoperatively acquired computed tomography (CT) in all patients. A rad-score was generated by using the least absolute shrinkage and selection operator (lasso) logistic model. Kaplan-Meier and Cox’s hazard regression analyses were used to evaluate the prognostic significance of the rad-score. Final nomograms predicting recurrence and survival of solitary HCC patients were established based on the rad-score and clinicopathological factors. C-index and calibration statistics were used to assess the performance of nomograms.

**Results:**

Six potential radiomics features were selected out of 110 texture features to formulate the rad-score. Low rad-score positively correlated with aggressive tumor phenotypes, like larger tumor size and vascular invasion. Meanwhile, low rad-score was significantly associated with increased recurrence and reduced survival. In addition, multivariate analysis identified the rad-score as an independent prognostic factor (recurrence: Hazard ratio (HR): 2.472, 95% confident interval (CI): 1.339–4.564, *p* = 0.004;survival: HR: 1.558, 95%CI: 1.022–2.375, *p* = 0.039). Notably, the nomogram integrating rad-score had a better prognostic performance as compared with traditional staging systems. These results were further confirmed in the validation cohort.

**Conclusions:**

The preoperative CT image based rad-score was an independent prognostic factor for the postoperative outcome of solitary HCC patients. This score may be complementary to the current staging system and help to stratify individualized treatments for solitary HCC patients.

**Electronic supplementary material:**

The online version of this article (10.1186/s12885-018-5024-z) contains supplementary material, which is available to authorized users.

## Background

Hepatocellular carcinoma (HCC) is the fifth most common cancer and the second most common cause of cancer-related death worldwide [1]. Current HCC staging systems, like Barcelona Clinic Liver Cancer (BCLC) staging system, indicate that hepatectomy is a potentially curative treatment for patients with early-stage HCC [2]. However, postoperative recurrence is high, with 5-year rates reaching 70% [3, 4], suggesting that even in the same early-stage, patients have a diverse postoperative prognosis. Thus, the current staging systems still need improvement, for example, incorporating new risk factors for a better stratification of postoperative outcome. In fact, traditional staging systems mainly consist of pathological factors, like tumor size and vascular invasion, while tremendous information in preoperative computed tomography (CT) or magnetic resonance imaging (MRI) reflecting tissue intrinsic characters and heterogeneity [5–8] remains untapped. Recently, it has been reported that various imaging features were associated with pathological features and prognosis of the tumor and complementary to current staging systems, like rectal cancer and bladder cancer [9, 10]. As such, new prognostic factors, like those derived from CT and MRI images, to identify patients with high risk of postoperative recurrence and death are urgently needed, which could help to select patients who are more likely to benefit from surgery.

Radiomics, an emerging and promising field, hypothesizes that medical images, including CT and MRI, could provide vivid and crucial information on tumor [11]. By converting medical images into high-dimensional, mineable and quantitative features via high-throughput data extraction, radiomics method provides an unprecedented opportunity to improve decision-support in oncology at low cost and noninvasively. Currently, image examinations are routinely conducted for cancer patients, including HCC [12]. Compared to developing new molecular biomarkers, radiomics method may not require additional physical or molecular tests and thus not increase the economic burden of patients. In addition, previous studies have demonstrated that quantitative radiomics features were associated with clinical prognosis and underlying genomic patterns across a range of cancer types, such as non-small cell lung cancer [13] and advanced nasopharyngeal carcinoma [14].

In HCC, contrast-enhanced computed tomography (CECT) has been widely used in the diagnosis due to its high specificity and sensitivity [12]. Meanwhile, it had been reported that the characteristics of tumor CT images were associated with gene expression profiles, pathological features, and prognosis of HCC [11, 15–17]. As far as we are concerned, image features could be divided into semantic features and agnostic features. Semantic features are commonly used in the radiology lexicon to describe regions of interest, including internal arteries, hypodense halos and so on, while agnostic features, like texture features, attempt to capture lesion heterogeneity though quantitative descriptors [11, 14, 18, 19]. Previous studies preferred the clinical application of semantic features, as they were easy to acquire. Recently, growing concerns have been paid on the potential clinical application of agnostic features. For instance, Fu et al. investigated the prognostic significance of CT image texture features for advanced HCC patients receiving TACE (transarterial chemoembolization) [15]. Another study has suggested that texture analysis was promising for HCC patient stratification for determining the suitability of liver resection vs. TACE [11]. Furthermore, texture analysis has been reported for the potential for predicting postoperative hepatic insufficiency and assessing fibrosis [20]. However, the prognostic significance of radiomics feature has been rarely investigated in HCC patients receiving hepatectomy.

In this study, we aimed at developing a rad-score derived from the preoperative CECT of solitary HCC patients, based on the assumption that such rad-score may help to identify patients who were at high risk of postoperative recurrence and death and improve clinical decision making for solitary HCC patients.

## Methods

### Patient selection and data collection

Patient recruitment, as well as the inclusion and exclusion criteria, were presented in Additional file 1: Figure S1. A total of 319 patients were enrolled and randomly divided into a training cohort (*n* = 212) and validation cohort (*n* = 107). The pathological diagnoses on all cases were reviewed and confirmed independently by two expert pathologists.

Baseline clinicopathological data were derived from medical records. Tumor differentiation was graded by the Edmondson grading system [21]. Postoperative follow-up strategy and treatment strategy were according to a uniform guideline as we previously described [22, 23], and were listed in the Additional file 2. Ethical approval was obtained from the institutional review board of Zhongshan Hospital, and the informed consent requirement was waived. Time to recurrence (TTR) was defined as the interval between surgery and recurrence or the last observation for surviving patients without recurrence. Overall survival (OS) was defined as the interval between surgery and death or the last observation for surviving patients. The data were censored at the last follow-up for living patients.

### Quantitative imaging characteristics

CT protocols and details of texture features are described in Additional file 2. Arterial phase CECT data were retrieved from the institution archive in dicom format and loaded to a personal laptop for further textural analysis. In this study, a total of 110 candidate radiomics features were generated from one image by using an in-house algorithm implemented in Matlab 2016a (MathWorks, Natick, MA, USA). For texture analysis, a region of interest (ROI) was delineated initially around the tumor outline of the largest cross-sectional area. Details of texture feature extraction are presented in Additional file 3: Figure S2.

### Inter-observer and intra-observer reproducibility of radiomics feature extraction

Sixty images were randomly chosen for evaluating the inter-observer reproducibility of the radiomics feature. All these images were reviewed by two radiologists with 10 (reader 1) and 5 years (reader 2) experience in abdominal CT interpretation. To assess the intra-observer reproducibility, reader 1 repeated the generation of texture features twice in a 1-week period followed the same procedure.

A two-way random, single measure (absolute agreement) intraclass correlation coefficients (ICC) was used to assess the differences between the features generated by reader 1 (first time) and those by reader 2, as well as between the twice-generated features by reader 1. An ICC value below 0.40 was considered poor reliability, fair for values between 0.41 and 0.59, good for values between 0.60 and 0.74, and excellent for values between 0.75 and 1.00. This is a descriptive statistic can be used when quantitative measurements are made on units that are organized into groups. It describes how strongly units in the same group resemble each other. Previously, it has been reported as a reliable method to evaluate the reproducibility of data [24, 25] and has been used in the radiomics research [26].

### Feature selection and rad-score building

According to the Harrell’s guideline, the number of events should exceed the number of included covariates by at least 10 times in a multivariate analysis. Therefore, in our study, the least absolute shrinkage and selection operator (lasso) method combined with logistic regression [27], was used to select the most useful features in the training cohort. This method minimized a log partial likelihood subject to the sum of the absolute values of the parameters being bounded by a constant:

$$ \widehat{\upbeta}=\mathrm{argmin}\ \mathrm{\ell}\left(\upbeta \right),\mathrm{subject}\ \mathrm{to}\sum \left|{\upbeta}_{\mathrm{j}}\right|\le \mathrm{s} $$

where, β ^ is the obtained parameters, l(β) is the log partial likelihood of the logistic regression model, s>0 is a constant.

As a benefit of the absolute constraint, the lasso method shrinks coefficients and changes some coefficients to zero [28]. Therefore, it can be used for the feature reduction and selection. In this study, the standardized constraint parameter s was set as 0.00013868 and lasso selected 6 nonzero coefficients ($\widehat{\upbeta}$). Then, the logistic regression model was obtained with its outcome being the hazard rate at the fifth year after operation for individuals. The R software and “glmnet” package (R foundation for Statistical computing, Vienna, Austria, URL: http://www.R-project.org, 2016) were used for the lasso logistics regression model analysis.

### Statistical analysis

Statistical analyses were performed using SPSS software (20.0; SPSS, Inc., Chicago, IL, USA) and R software (R Foundation for Statistical Computing, Vienna, Austria) with the “rms” package (R Foundation for Statistical Computing, Vienna, Austria). Continuous variables were compared using the Mann-Whitney U, while category variables were compared using Chi-squared or Fisher’s exact tests. X-tile (Yale University, New Haven, CT, USA) software was used to determine the optimal cut-off value of the rad-score, which is a graphical method that illustrates the presence of substantial tumor subpopulations and shows the robustness of the relationship between a biomarker and outcome by construction of a two-dimensional projection of every possible subpopulation [29, 30]. Survival curves were depicted using Kaplan–Meier analysis (log-rank test). The Cox’s proportional hazards regression model was applied for univariate and multivariate analyses. “Rms” package was used to build nomogram models. The Harrell’s concordance index (C-index) and calibration curves were used to evaluate the nomogram models [31]. Details of nomogram models were listed in the Additional file 2. A two-sided value of *p* < 0.05 was considered statistically significant.

## Results

### Clinical characteristics of the patients

No significant differences in clinicopathological features were observed between the two cohorts (Table 1). All patients were solitary HCC and received R0 resection. The mean follow-up time in training and validation cohorts was 52.7 ± 21.6 months and 54.5 ± 22.1 months, respectively. Overall survival rates at 1, 3, and 5 years after operation was 87, 76 and 69% for training cohort and 88, 75 and 72% for validation cohort, respectively.

**Table 1:** Clinicopathological features of HCC patients in training and validation cohorts

Variable	Training cohort	Validation cohort	*p*
Median(range) age, y	55(24–85)	55(13–83)	0.93
Gender (male/female)	175/37	88/19	0.95
Tumor size(≤3,> 3), cm	76/133	38/69	0.82
Vascular invasion(Present/Absent)	22/190	12/95	0.82
Microvascular invasion(Present/Absent)	68/144	29/78	0.36
Tumor differentiation (III-IV/I-II)	16/193	6/101	0.46
Liver cirrhosis(Present/Absent)	166/46	87/20	0.53
HbsAg (Positive/Negative)	178/34	85/22	0.32
Tumor encapsulation (Present/ Absent)	112/110	59/48	0.70
Preoperative blood test			
DBIL(mean ± SD), μmol/L	6.9 ± 11.9	7.3 ± 16.4	0.45
TBIL(mean ± SD), μmol/L	14.8 ± 14.5	15.04 ± 19.2	0.94
ALT(mean ± SD), U/L	40.1 ± 31.3	46.1 ± 69.2	0.91
AST(mean ± SD), U/L	38.7 ± 27.3	453.6 ± 66.3	0.67
AFP(mean ± SD), ng/ml	4460 ± 13,186	3800 ± 12,646	0.60
ALB(mean ± SD), g/L	40.7 ± 7.0	39.8 ± 3.6	0.06
GGT(mean ± SD), U/L	84.3 ± 98.8	80.8 ± 83.5	0.38

*Abbreviations*: *ALB* Albumin, *ALT* Alanine aminotransferase, *AST* Aspartate aminotransferase, *DBIL* Direct Bilirubin, *TBIL* Total bilirubin, *GGT*, γ-Glutamyltransferase, *AFP* Alpha-fetoprotein

### Results of inter-observer and intra-observer reproducibility of radiomics feature extraction

Satisfactory inter- and intra-observer reproducibility of the texture feature extraction was achieved. The reproducibility of radiomics feature extraction was good between the two readers (ICC range: 0.71–0.95) or between reader 1’s first and second-extracted features (ICC range: 0.83–0.99). These results suggested that our radiomics feature values were highly reproducible.

### Development of the rad-score and its association with clinicopathological features

Six features were selected out of 110 texture features by using the lasso-logistic selection of the basis of 212 patients in the training cohort (Additional file 4: Figure S3). The rad-score calculation formula consisting of these features was presented in Additional file 2. All the coefficients in the equation are from lasso-logistic regression. Determined by X-tile software, the optimal cut-off for rad-score was 4.32 (Rad-score range: Training cohort: 1.70–22.3; Validation cohort: 2.1–29.2). Accordingly, patients were divided into high (> 4.32) and low (≤ 4.32) groups.

Further investigation was performed to assess the association between the rad-score and clinicopathological features in the training cohort (Additional file 5: Table S1). Patients with low rad-score were positively associated with high preoperative alpha-fetoprotein (AFP) level (*p* < 0.001), larger tumor size (*p* < 0.001), presence of vascular invasion (*p* = 0.009), advanced TNM stage (*p* = 0.015) and BCLC stage (*p* = 0.020), suggesting that low rad-score may indicate tumor aggressiveness.

### Low rad-score correlated with poor survival in solitary HCC patients

**Fig. 1:**
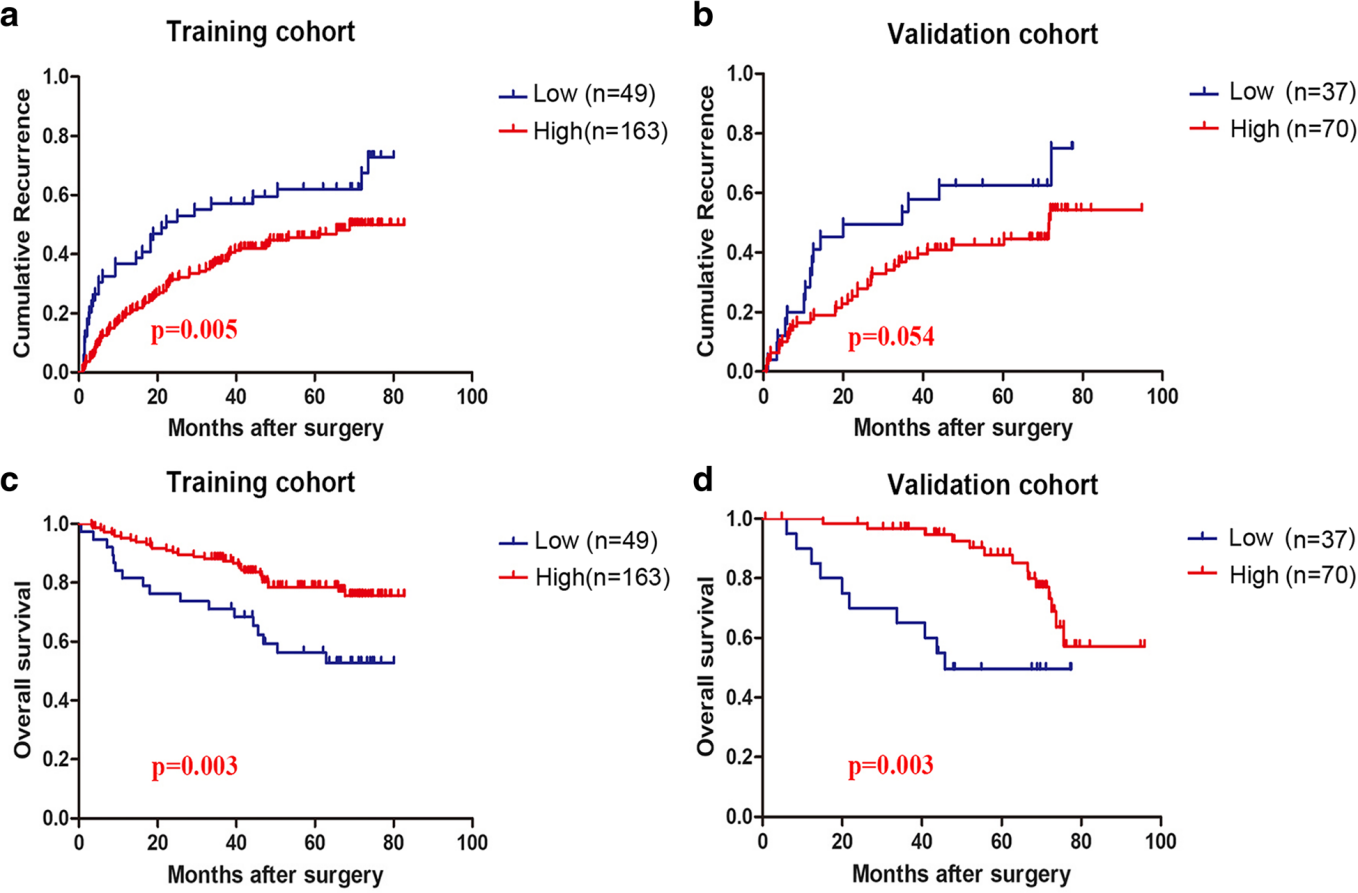
Prognostic significance of rad-score for solitary HCC patients. **a** Training cohort: median TTR [95%CI] for low rad-score [*n* = 49] versus high rad-score [*n* = 163]: 38 [28.2–47.1] versus 53 [48.0–58.4] months; *p* = 0.005. **b** Validation cohort: *p* = 0.054. **c** Training cohort: median OS [95%CI] for low rad-score [n = 49] versus high rad-score [n = 163]: 54.9[45.4–64.5] versus 70.5 [66.6–74.5] months; *p* = 0.003. **d** Validation cohort: median OS [95%CI] for low rad-score [*n* = 37] versus high rad-score [*n* = 70]: 50.9[38.5–63.3] versus 82.2 [75.6–88.8] months; *p* = 0.003

In the training cohort, low rad-score were significantly associated with shorter TTR (median TTR [95% confident interval (CI)] for low [*n* = 49] versus high rad-score [*n* = 163]: 38 [28.2–47.1] versus 53 [48.0–58.4] months; *p* = 0.005, Fig. 1a). In the validation cohort, no significance was observed in recurrence between the two groups with the *p* value of 0.054 (Fig. 1b), suggesting that the rad-score was slightly over-fitted to the training cohort. As for OS, low rad-score significantly correlated with shorter postoperative survival in both training cohort (median OS [95% CI] for low [n = 49] versus high rad-score [n = 163]: 54.9[45.4–64.5] versus 70.5 [66.6–74.5] months; *p* = 0.003, Fig. 1c) and validation cohort (median OS [95%CI] for low [*n* = 37] versus high [*n* = 70]: 50.9[38.5–63.3] versus 82.2 [75.6–88.8] months; *p* = 0.003, Fig. 1d).

Multivariate analyses suggested that rad-score was an independent prognostic factor of recurrence in the training cohort (Hazard ratio (HR): 2.472, 95%CI: 1.339–4.564, *p* = 0.004, Table 2). As for OS, the rad-score (HR: 1.558, 95%CI: 1.022–2.375, *p* = 0.039, Table 3) was also identified as an independent prognostic factor in the training cohort. Similar results were observed in the validation cohort (recurrence: HR: 1.890, 95%CI: 1.04–3.436, *p* = 0.036, Table 2; survival: HR: 3.236, 95%CI: 1.416–7.407, *p* = 0.005, Table 3).

**Table 2:** Uni-and Multivariate analyses of predictors of postoperative recurrence in training and validation cohorts

Variables		Training cohort						Validation cohort		
		Univariate			Multivariate			Multivariate		
		HR	95%CI	*p*	HR	95%CI	*p*	HR	95%CI	*p*
Gender	Male	1.131	0.672–1.903	0.642	\	\	\	\	\	\
	Female									
Tumor size	≤3 cm	1.831	1.190–2.817	0.006	\	\	0.193	\	\	0.167
	> 3 cm									
Vascular invasion	Present	2.364	1.341–4.166	0.003	2.602	1.408–4.807	0.002	\	\	0.141
	Absent									
Microvascular invasion	Present	1.285	0.858–1.926	0.223	\	\	\	\	\	\
	Absent									
Differentiation	III-IV	1.242	0.871–1.772	0.231	\	\	\	\	\	\
	I-II									
Liver Cirrhosis	YES	1.076	0.89–1.301	0.451	\	\	\	\	\	\
	NO									
HBsAg	Positive	1.292	0.747–2.233	0.359	\	\	\	\	\	\
	Negative									
Tumor encapsulation	Present	1.533	1.032–2.277	0.034	1.637	1.074–2.494	0.022	\	\	0.975
	Absent									
Rad-score	≤4.32	1.779	1.174–2.703	0.007	1.558	1.022–2.375	0.039	1.890	1.041–3.436	0.036
	> 4.32									
DBIL, μmol/L		1.002	0.986–1.019	0.797	\	\	\	\	\	\
TBIL, μmol/L		1.003	0.99–1.015	0.691	\	\	\	\	\	\
ALT, U/L		1.001	0.998–1.004	0.624	\	\	\	\	\	\
AST, U/L		1.001	0.999–1.004	0.339	\	\	\	\	\	\
AFP, ng/ml		1.000	1.000–1.000	0.494	\	\	\	\	\	\
ALB, g/L		1.020	0.986–1.055	0.253	\	\	\	\	\	\
GGT, U/L		1.002	1.001–1.004	0.002	1.002	1.000–1.003	0.017	\	\	0.426

*Abbreviations*: *ALB* Albumin, *ALT* Alanine aminotransferase, *AST* Aspartate aminotransferase, *DBIL* Direct Bilirubin, *TBIL* Total bilirubin; *GGT* γ-Glutamyltransferase, *AFP* Alpha-fetoprotein

**Table 3:** Uni-and Multivariate analyses of predictors of postoperative survival in training and validation cohorts

Variables		Training cohort						Validation cohort		
		Univariate analysis			Multivariate analysis			Multivariate analysis		
		HR	95%CI	*p*	HR	95%CI	*p*	HR	95%CI	*p*
Gender	Male	0.931	0.451–1.923	0.847	\	\	\			
	Female									
Tumor size(cm)	≤3	2.822	1.406–5.650	0.004	1.910	0.897–4.070	0.035	\	\	0.139
	> 3									
Vascular invasion	Present	3.52	1.701–7.287	0.001	1.794	0.797–4.037	0.008	\	\	0.982
	Absent									
Micro-vascular invasion	Present	1.084	0.574–2.051	0.803	\	\	\	\	\	\
	Absent									
Differentiation	III-IV	1.515	0.886–2.592	0.129	\	\	\	\	\	\
	I-II									
Liver Cirrhosis	Yes	1.113	0.838–1.479	0.458	\	\	\	\	\	\
	No									
HBsAg	Present	1.557	0.662–3.664	0.31	\	\	\	\	\	\
	Absent									
Tumor encapsulation	Absent	2.105	1.155–3.846	0.015	2.326	1.272–4.255	0.006	\	\	0.394
	Present									
Rad-score	≤4.32	2.387	1.321–4.310	0.004	2.283	1.261–4.132	0.006	3.236	1.416–7.407	0.005
	> 4.32									
DBIL, μmol/L		1.015	0.998–1.031	0.084	\	\	\	\	\	
TBIL, μmol/L		1.012	0.998–1.026	0.102	\	\	\	\	\	\
ALT, U/L		0.998	0.991–1.005	0.57	\	\	\	\	\	\
AST, U/L		1.000	0.995–1.005	0.946	\	\	\	\	\	\
AFP, ng/ml		1.000	1.000–1.000	0.189	\	\	\	\	\	\
ALB, g/L		1.020	0.964–1.079	0.495	\	\	\	\	\	\
GGT, U/L		1.004	1.003–1.006	< 0.0001	1.004	1.002–1.006	< 0.0001	\	\	0.249

*Abbreviations*: *ALB* Albumin, *ALT* Alanine aminotransferase, *AST* Aspartate aminotransferase, *DBIL* Direct Bilirubin, *TBIL* Total bilirubin, *GGT*, γ-Glutamyltransferase, *AFP* alpha-fetoprotein

All these results demonstrated that rad-score was an independent prognostic factor of postoperative recurrence and survival for solitary HCC patients. Patients with low rad-score have a higher recurrence rate and poorer survival.

### The performance of rad-score based prognostic nomograms

**Fig. 2:**
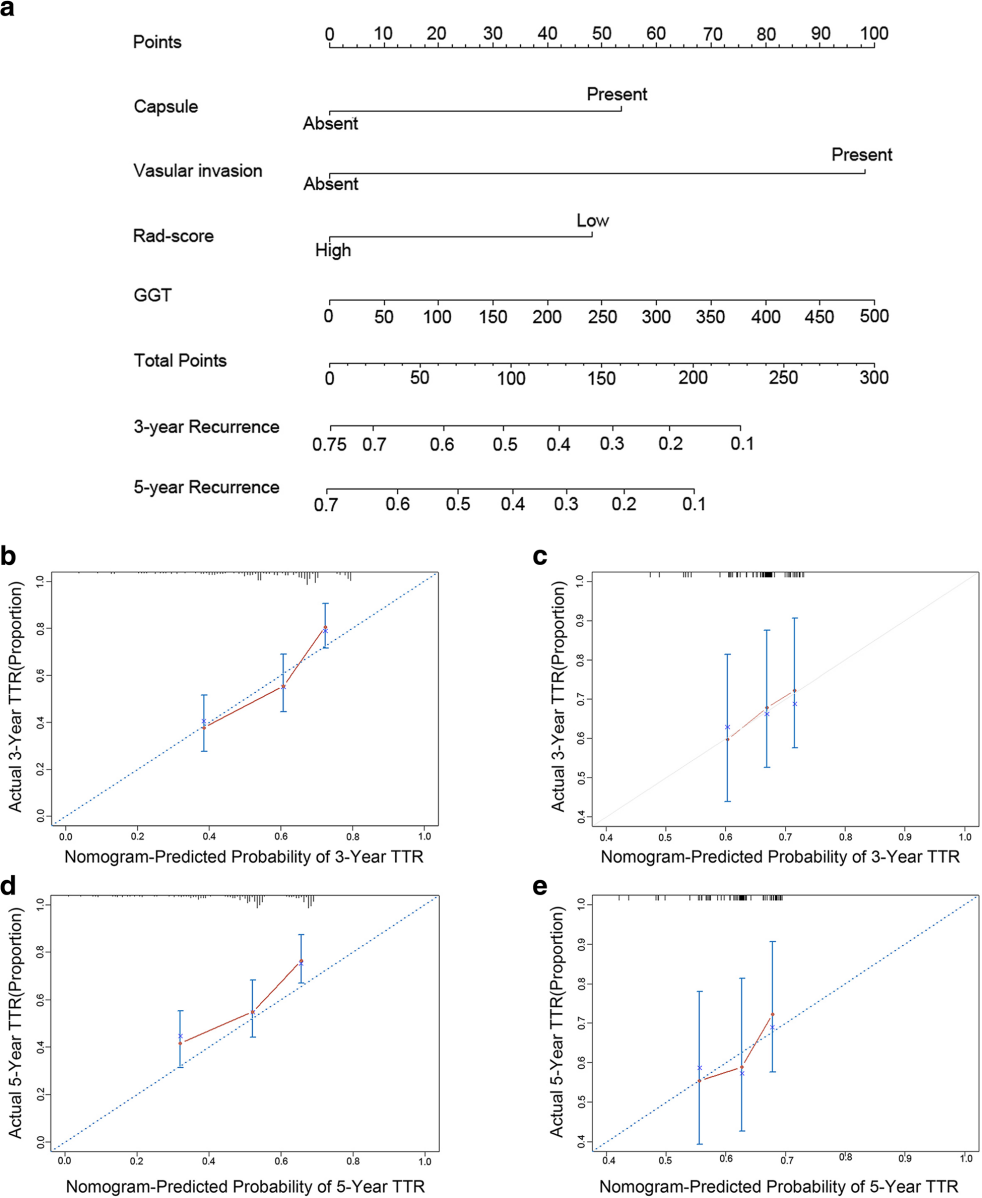
Development of rad-score based nomograms and calibration curves of the rad-score based nomogram for recurrence in both training and validation cohorts. **a** The prognostic nomogram for recurrence. **b** Calibration curves for 3 years TTR in the training cohort. **c** Calibration curves for 3 years TTR in the validation cohort. **d** Calibration curves for 5 years TTR in the training cohort. **e** Calibration curves for 5 years TTR in the validation cohort

Based on the results of multivariate analysis, rad-score based nomogram predicting postoperative recurrence (Fig. 2a) of solitary HCC patients was established. In the nomogram model, each factor was ascribed a weighted point that implied a risk of recurrence or survival. For example, low rad-score was ascribed 20 points (on a scale of 0–100 points) in nomogram for postoperative survival. Each patient with a high total score had a worse prognosis, namely higher risk of recurrence or death. C-index was used to evaluate the predictive accuracy (discrimination) of the rad-score based nomograms, which was 0.639 (95% CI: 0.577–0.701, Table 4) for the nomogram of recurrence and 0.714 (95% CI: 0.635–0.793, Table 4) for the nomogram of survival in the training cohort. In the validation cohort, the C-index was 0.587(95% CI: 0.479–0.695, Table 4) for nomogram of recurrence, and the C-index was 0.71 (95% CI: 0.602–0.808, Table 4) for nomogram of survival. In addition, 50-sample bootstrapped calibration plots revealed the good predictive accuracy of the nomogram for the prediction of 3- (Fig. 2b, c) and 5- (Fig. 2d, e) year recurrence rate in the training and validation cohorts.

**Table 4:** C-indices of rad-score based nomograms, clinicopathological nomograms and traditional staging systems

	Training cohort				Validation cohort			
	TTR		OS		TTR		OS	
	C-index	95%CI	C-index	95%CI	C-index	95%CI	C-index	95%CI
Rad-score	0.563	0.516–0.610	0.541	0.488–0.594	0.555	0.491–0.619	0.629	0.529–0.729
Rad-score based nomograms	0.639	0.577–0.701	0.714	0.635–0.793	0.587	0.479–0.695	0.710	0.602–0.818
Clinical-pathological nomograms	0.633	0.571–0.695	0.554	0.485–0.623	0.601	0.592–0.610	0.642	0.532–0.752
Rad-score-TNM nomogram	0.584	0.531–0.637	0.626	0.549–0.703	0.601	0.530–0.672	0.617	0.507–0.727
Rad-score-BCLC nomogram	0.580	0.526–0.634	0.627	0.549–0.705	0.601	0.523–0.679	0.622	0.509–0.735
Traditional staging system								
TNM	0.552	0.513–0.581	0.575	0.515–0.635	0.553	0.499–0.607	0.521	0.453–0.589
BCLC	0.547	0.506–0.588	0.574	0.511–0.637	0.548	0.485–0.611	0.506	0.420–0.592
JIS	0.554	0.508–0.600	0.601	0.533–0.669	0.564	0.503–0.625	0.574	0.504–0.644
HKLC	0.575	0.529–0.631	0.628	0.620–0.636	0.582	0.507–0.657	0.596	0.487–0.701

*Abbreviation*: *TNM* Tumor–node–metastasis, *BCLC* Barcelona Clinic Liver Cancer stage system, *JIS* Japan Integrated Staging, *HKLC* Hong Kong Liver Cancer staging score

**Fig. 3:**
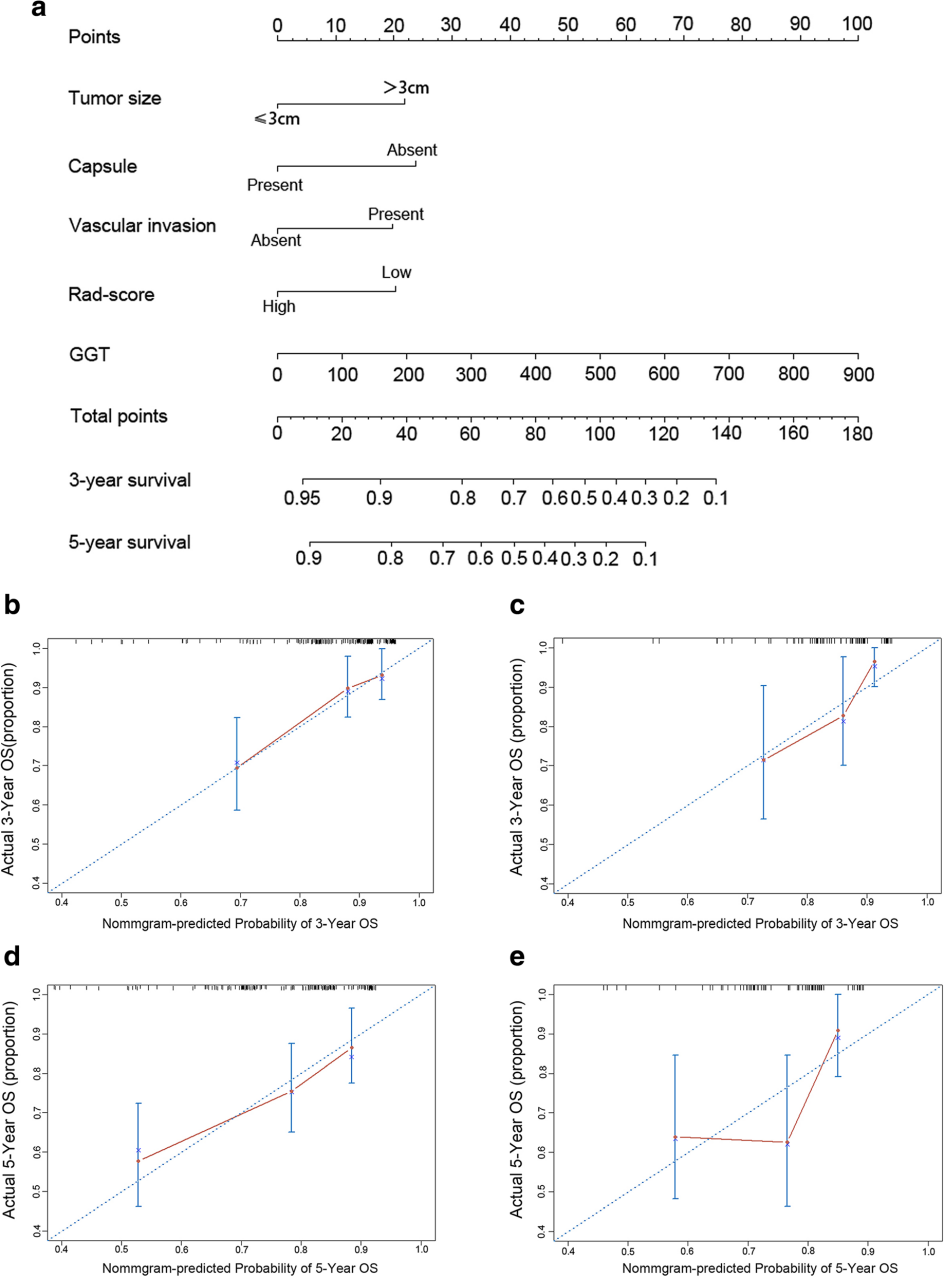
Development of rad-score based nomograms and calibration curves of the rad-score based nomogram for OS in both training and validation cohorts. **a** The prognostic nomogram for postoperative survival. **b** Calibration curves for 3 years OS in the training cohort. **c** Calibration curves for 3 years OS in the validation cohort. **d** Calibration curves for 5 years OS in the training cohort. **e** Calibration curves for 5 years OS in the validation cohort

Similarly, rad-score based nomogram prediction postoperative survival of solitary HCC patients was developed (Fig. 3a). Good predictive accuracy of 3- (Fig. 3b, c) and 5-(Fig. 3d, e) year survival rate was also observed in both training and validation cohorts.

Indeed, the Hosmer-Lemeshow test yielded no significant difference between the predictive calibration curve and the ideal curve for postoperative recurrence and survival prediction in both training and validation datasets. These results indicated that two nomograms could predict postoperative recurrence and survival effectively.

### Comparison between the rad-score based nomograms and traditional staging systems

Previously, several traditional staging systems have been proposed for patients with HCC, including 7th edition of the American Joint Committee on Cancer (AJCC) TNM staging criteria, BCLC staging system [32], Japan Integrated Staging (JIS) [33] score and Hong Kong Liver Cancer (HKLC) staging score [34]. In the training cohort, the C-index of these staging systems in predicting postoperative survival was 0.575 (95% CI: 0.515–0.635) for AJCC staging system, 0.574(95% CI: 0.511–0.637) for BCLC staging system, 0.601(95% CI: 0.533–0.669) for JIS staging system and 0.628(95% CI: 0.548–0.708) for HKLC staging system, respectively (Table 4). When being compared to C-indices of our new nomogram including the rad-score, the C-indices of these staging systems were significantly lower in both training and validation cohorts. As for recurrence, the C-index of four staging systems was 0.552 (95% CI: 0.513–0.581) for AJCC TNM staging system, 0.547 (95% CI: 0.506–0.588) for BCLC staging system, 0.554 (95% CI: 0.508–0.600) for JIS staging system and 0.575 (95% CI: 0.529–0.631) for HKLC staging system, respectively, significantly lower than the C-index of our nomogram including the rad-score in both training and validation cohorts (Table 4). All these results suggested that our rad-score based nomograms had a better discrimination performance than traditional staging system for solitary HCC patients.

### Assessment of incremental value of rad-score

To investigate the incremental value of rad-score in individual postoperative recurrence and survival prediction, we compared the discrimination performance of clinicopathological nomograms and rad-score based nomograms. The clinicopathological nomograms were established based on independent clinicopathological risk factors, with the C-index of 0.633 (95% CI: 0.571–0.695) for recurrence and 0.554 (95% CI: 0.485–0.623) for postoperative survival in the training cohort. The discrimination performance of the nomogram improved when the rad-score was integrated (recurrence: C-index, 0.639, 95%CI: 0.577–0.701; survival: C-index, 0.714, 95%CI: 0.635–0.793), significantly higher than the discrimination performance of clinicopathological nomogram in the training cohort (Table 4). In the validation cohort, similar results were observed for postoperative survival. The C-index of clinicopathological nomogram was 0.642 (95%CI: 0.532–0.752), while the C-index (0.710, 95%CI: 0.602–0.818) improved after incorporating the rad-score into nomogram (Table 4). These results suggested that the rad-score was a good complementary to clinicopathological factors in individual postoperative recurrence and survival prediction.

The similar analysis was performed for traditional staging systems. An improvement in evaluating postoperative recurrence and survival was observed after combining the rad-score with the TNM staging system and BCLC staging system (Table 4). Hence, the rad-score is complementary to the TNM and BCLC staging system, demonstrating the valuable prognostic role of rad-score.

## Discussion

In this study, a multi-CT-texture feature based rad-score was proposed, which successfully stratified patients into groups with significant differences in TTR and OS, and may be complementary to traditional staging systems.

Radiomics, a promising field of oncological research, assume that image features could predict the prognosis of patients, as they are associated with tumor biological characteristics [11, 35]. Previous studies have supported this hypothesis [17, 36]. For instance, Banerjee et al. proposed an image features of venous invasion, consisting of three semantic features (internal arteries, hypodense halo, and tumor liver difference), were closely associated with early recurrence and poor survival for HCC [37]. Similarly, the rad-score identified in our study was closely associated with pathological factors of HCC, like larger tumor size and vascular invasion and could be predictive of recurrence and survival.

Previously, several staging systems have been proposed for HCC patients, including TNM, BCLC, and HKLC [38]. Our rad-score based nomograms yielded a better discriminative ability than these traditional staging systems for solitary HCC patients. In addition, our results suggested that the rad-score could complement the TNM and BCLC staging systems in prognostic stratification as the C-index value increased when the rad-score was added to them. This incremental ability indicated the clinical importance of our finding for solitary HCC patients.

In our study, lasso-logistic regression model was performed to select texture features to establish the rad-score, as features obtained from lasso were generally accurate and the regression coefficients of most features were shrunk toward zero during overfitting [39], making the model easier to interpret and allowing the identification of the most valuable features [40]. Indeed, this method had been widely used in similar studies [14, 19].

Of note, the C-index values were relatively low for traditional staging systems, this phenomenon may be attributed to the study design. In our study, only solitary HCC patients were included. According to the traditional staging systems, these patients belong to the early or intermediate stages and are appropriate for surgery. Although they share the same or similar stage, a great deal of heterogeneity exists among them and they have a diverse postoperative prognosis. Thus, traditional staging systems could not actually predict recurrence and survival for these patients. In addition, the rad-score proposed also shared a relatively low C-index, but this couldn’t affect the clinical significance of rad-score, as it could stratify these patients into groups with different prognosis and improved the prognostic performance of traditional staging systems when being added into them for these patients.

The current study had several limitations. On one hand, the data in this study were derived from only one hepatobiliary center. On the other hand, only solitary HCC patients were included in this study, which may influence the generalization of the conclusion. In addition, this is a retrospective research. Therefore, further perspective multicenter analyses including HCC patients as various tumor stages were needed to validate the prognostic significance of this rad-score.

## Conclusions

In summary, a rad-score derived from CT texture features was proposed in this study, which was an independent prognostic factor for tumor recurrence and survival of solitary HCC patients. In addition, this image score was complementary to the current staging systems of HCC patients. Finally, prognostic nomograms combining this score and clinicopathological features were proposed, which outperformed traditional staging systems and provided a convenient way to predict prognosis for solitary HCC patients, and may influence decision-making on the possible benefit of surgery.

## Additional files

Additional file 1: **Figure S1.** Flow chart of patient selection. (TIF 364 kb)

Additional file 2: Details of methodology and results which were not shown in the manuscript. (DOCX 25 kb)

Additional file 3: **Figure S2.** The process of radiomics and the use of radioimics in decision support. (a) The acquisition of high-quality images. (b) A region of interest (ROI) was identified by experienced radiologists. (c) Texture features were extracted from ROI. (d) These features were mined to develop prognostic models for clinical outcomes. (TIF 1127 kb)

Additional file 4: **Figure S3.** Texture feature selection using the least shrinkage and selection operator (lasso) binary logistic regression model. (a) Tuning parameter (λ) selection in the lasso model using 10-fold cross-validation via minimum criteria. The area under the receiver operating characteristic curve (AUC) was plotted versus log (λ). Dotted vertical lines were drawn at the optimal values by using the minimum criteria and the λ standard error of the minimum criteria (the 1-SE criteria). A value of 0.00013868, with log (λ) -3.858 was chosen (1-SE criteria) according to 10-fold cross-validation. (b) Lasso coefficient profiles of the 110 texture features. A coefficient profile plot was produced against the log (λ) sequence. Vertical line was drawn at the value selected using 10-fold cross-validation, where optimal λ resulted in 6 nonzero coefficients. (TIF 17604 kb)

Additional file 5: **Table S1.** Association between rad-score and clinicopathological features in training cohort (DOCX 2509 kb)

## Abbreviations

AFP: Alpha-fetoprotein
AJCC: American Joint Committee on Cancer
ALB: Albumin
ALT: Alanine aminotransferase
AST: Aspartate aminotransferase
BCLC: Barcelona clinical liver cancer
CECT: Contrast-enhanced computed tomography
CI: Confidence interval
C-index: Harrell’s concordance index
CT: Computed tomography
DBIL: Direct bilirubin
GGT: γ-Glutamyltransferase
HCC: Hepatocellular carcinoma
HKLC: Hong Kong Liver Cancer
HR: Hazard ratio
ICC: Intraclass correlation coefficients
JIS: Japan Integrated Staging
Lasso: Least absolute shrinkage and selection operator
MRI: Magnetic resonance imaging
OS: Overall survival
Rad-score: Radiomics score
ROI: Region of interest
RVI: Radiogenomics venous invasion
TACE: Transarterial chemoembolization
TBIL: Total bilirubin
TNM: Tumor-node-metastasis
TTR: Time to recurrence
