# Supplementary figures and images for: Radiomics score: a potential prognostic imaging feature for postoperative survival of solitary HCC patients
Source: BMC Cancer. 2018 Nov 21;18:1148. doi: 10.1186/s12885-018-5024-z (PMC6249916; doi:10.1186/s12885-018-5024-z)

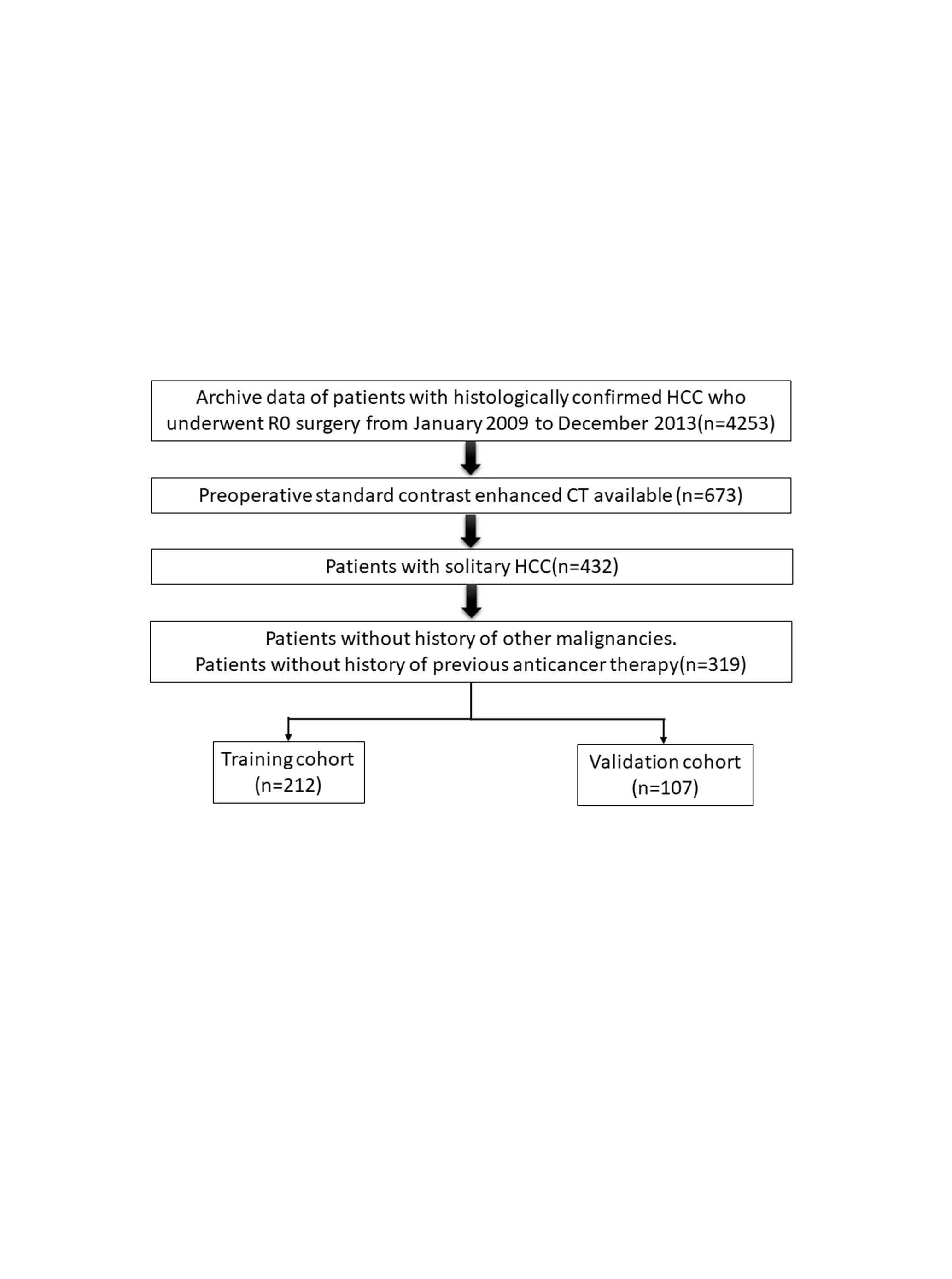

Supplement: Supplementary file 1 — Figure S1. Flow chart of patient selection. (TIF 364 kb) [file 12885_2018_5024_MOESM1_ESM.tif]

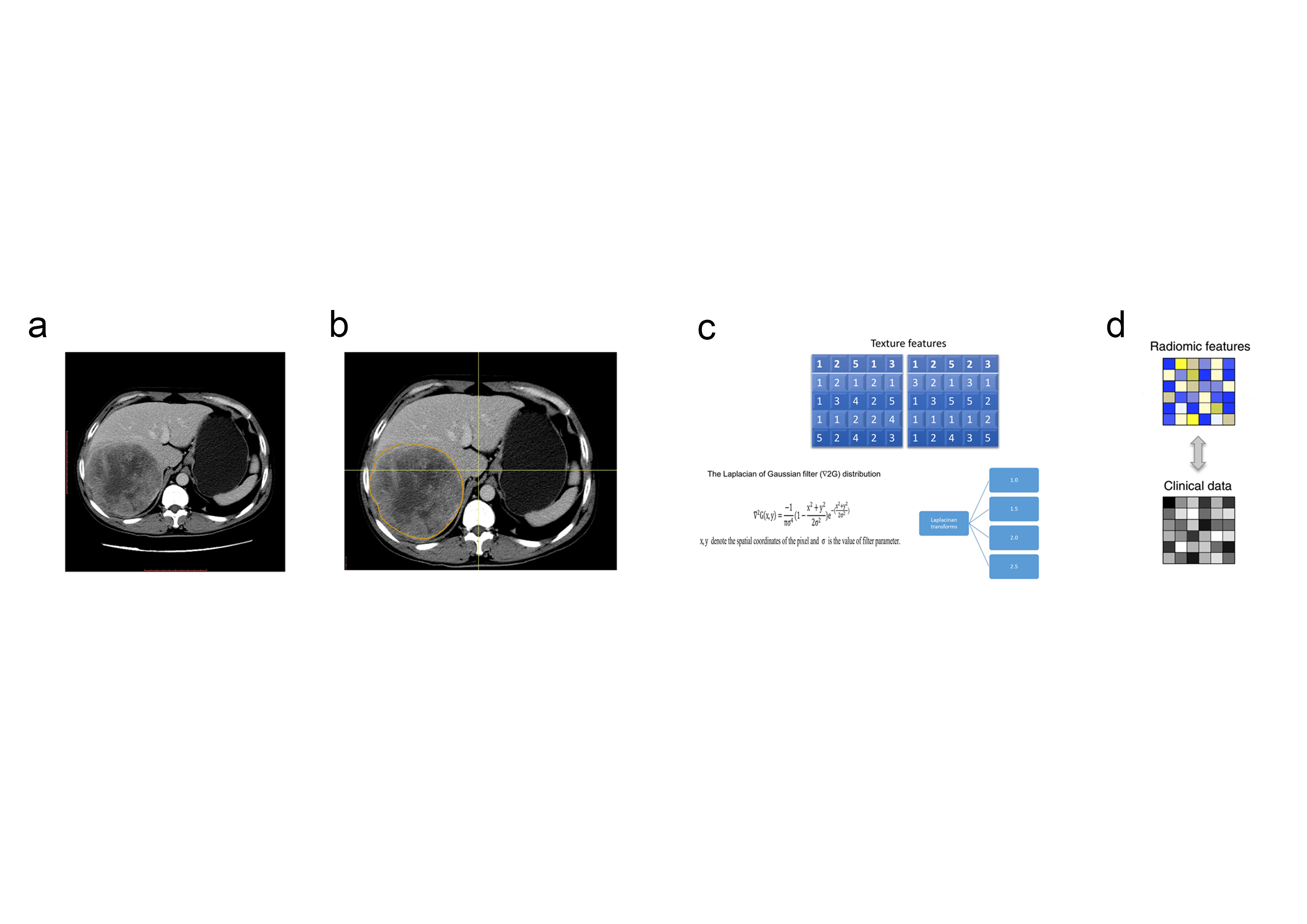

Supplement: Supplementary file 3 — Figure S2. The process of radiomics and the use of radioimics in decision support. (a) The acquisition of high-quality images. (b) A region of interest (ROI) was identified by experienced radiologists. (c) Texture features were extracted from ROI. (d) These features were mined to develop prognostic models for clinical outcomes. (TIF 1127 kb) [file 12885_2018_5024_MOESM3_ESM.tif]

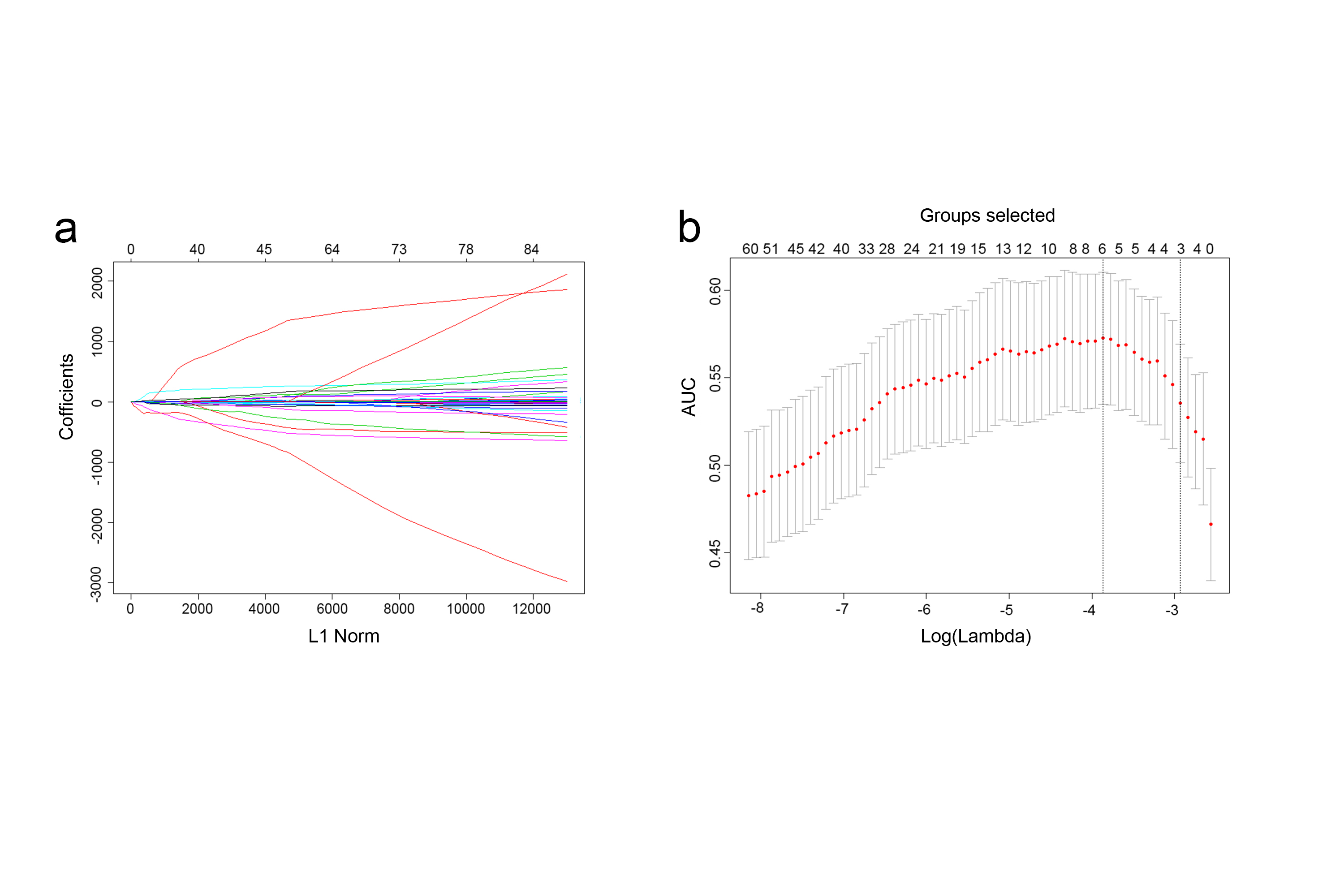

Supplement: Supplementary file 4 — Figure S3. Texture feature selection using the least shrinkage and selection operator (lasso) binary logistic regression model. (a) Tuning parameter (λ) selection in the lasso model using 10-fold cross-validation via minimum criteria. The area under the receiver operating characteristic curve (AUC) was plotted versus log (λ). Dotted vertical lines were drawn at the optimal values by using the minimum criteria and the λ standard error of the minimum criteria (the 1-SE criteria). A value of 0.00013868, with log (λ) -3.858 was chosen (1-SE criteria) according to 10-fold cross-validation. (b) Lasso coefficient profiles of the 110 texture features. A coefficient profile plot was produced against the log (λ) sequence. Vertical line was drawn at the value selected using 10-fold cross-validation, where optimal λ resulted in 6 nonzero coefficients. (TIF 17604 kb) [file 12885_2018_5024_MOESM4_ESM.tif]
