# Supplementary material for: Radiomics score: a potential prognostic imaging feature for postoperative survival of solitary HCC patients
Source: BMC Cancer. 2018 Nov 21;18:1148. doi: 10.1186/s12885-018-5024-z (PMC6249916; doi:10.1186/s12885-018-5024-z)
Supplement: Supplementary file 2 — Details of methodology and results which were not shown in the manuscript. (DOCX 25 kb) [file 12885_2018_5024_MOESM2_ESM.docx]

**Supplementary materials**

# Follow up strategy

All patients were monitored prospectively by serum alpha-fetoprotein (AFP), CEA, CA19-9, abdomen ultrasonography, and chest X-ray every 2–3 months in the first 2 years after surgery. Thereafter, these examinations were performed every 3–6 months. If a patient was suspected for recurrence, enhanced computed tomography and/or magnetic resonance imaging were performed to figure out whether recurrence had occurred. The diagnosis of recurrence was based on typical imaging appearance in computed tomography/magnetic resonance imaging with an elevated AFP level. After recurrence being confirmed, a second hepatectomy, radiofrequency ablation, chemotherapy, transcatheter arterial chemoembolization, or external radiotherapy were carried out according to the characteristics of tumor, like number, size, and location of the recurrent tumor.

# CT protocols

All patients underwent contrast-enhanced abdominal CT using one of the three multi-detector row CT (MDCT) systems (64 slice Light speed VCT, GE Medical systems, 64 slice CT, Siemens, and 16 slice CT, Siemens) before surgery. The acquisition parameters were as follows: 120kV; 160 mAs; 0.5- or 0.4-second rotation time; detector collimation: 64×2.5mm; field of review, 350×350mm; matrix, 512×512. After routine non-enhanced CT, arterial and portal venous-phase contrast-enhanced CT were performed after 22s and 60s delay, following intravenous administration of 90 - 100 ml of iodinated contrast material (Ultravist 370, Bayer Schering Pharma, Berlin, Germany) at a rate of 3.0 or 3.5ml/s with a pump injector (Ulrich CT Plus 150, Ulrich Medical, Ulm, Germany). Contrast-enhanced CT was reconstructed with reconstruction thickness of 5 mm.

# Radiomics feature extraction

Texture analysis was applied to the CT images using in-house texture analysis software with algorithms implemented in Matlab 2016a (Mathworks, Natick, USA). Arterial phase contrast-enhanced CT data was retrieved from the institution archive and loaded to CEER for textural analysis. A region of interest (ROI) was delineated initially around the tumor outline for the largest cross-sectional area. In total, 110 imaging texture features from the category of gray-level co-occurrence matrix (GLCM) were finally extracted from one single image.

1. **Image filtration:**

A Laplacian of Gaussian spatial band-pass filter (∇^2^G) was used to derive image features at different spatial scales by turning the filter parameter between 1.0 and 2.5 (1.0, 1.5, 2.0, 2.5).

The Laplacian of Gaussian filter (∇2G) distribution is given by

$$\nabla^{2}G\left( x,y \right)=\frac{-1}{\pi\delta^{4}}\left( 1-\frac{x^{2}+y^{2}}{2\sigma^{2}} \right)e^{-\left( \frac{x^{2}+y^{2}}{2\sigma^{2}} \right)}$$

x, y denote the spatial coordinates of the pixel and δ is the value of filter parameter.

1. **Feature generation**

A series of gray-level co-occurrence matrix (GLCM) texture features [[1-3](#_ENREF_1)] were generated from the image without or after filtration.

Gray-Level Co-Occurrence Matrix (GLCM) is a second-order statistical texture feature (**Table 1**), which is defined as a matrix Pδ (i,j) to indicate the relative frequency with intensity values of two pixels (i and j) at the distance of δ=1. x, y denote the spatial coordinates of the pixel. P(i,j) is the co-occurrence matrix by the δ.

**Table 1 Classification of GLCM texture features**

| **GLCM texture features** | |
| --- | --- |
| Autocorrelation_δ: [[2](#_ENREF_2)] | Maximum probability_δ: [[2](#_ENREF_2)] |
| Contrast_δ: matlab/[[1](#_ENREF_1), [2](#_ENREF_2)] | Sum of squares: Variance_δ[[1](#_ENREF_1)] |
| Correlation_δ: matlab[[1](#_ENREF_1), [2](#_ENREF_2)] | Sum average_δ[[1](#_ENREF_1)] |
| Correlation_δ: [[1](#_ENREF_1), [2](#_ENREF_2)] | Sum variance_δ[[1](#_ENREF_1)] |
| Cluster Prominence_δ: [[2](#_ENREF_2)] | Sum entropy_δ[[1](#_ENREF_1)] |
| Cluster Shade_δ: [[2](#_ENREF_2)] | Difference variance_δ[[1](#_ENREF_1)] |
| Dissimilarity_δ: [[2](#_ENREF_2)] | Difference entropy_δ[[1](#_ENREF_1)] |
| Energy_δ: matlab / [[1](#_ENREF_1), [2](#_ENREF_2)] | Information measure of correlation1_δ[[1](#_ENREF_1)] |
| Entropy_δ: [[2](#_ENREF_2)] | Informaiton measure of correlation2_δ[[1](#_ENREF_1)] |
| Homogeneity_δ: matlab | Inverse difference (INV) is homom_δ[[1](#_ENREF_1)] |
| Homogeneity_δ: [[2](#_ENREF_2)] | Inverse difference normalized (INN) _δ[[1](#_ENREF_1)] |
|  | Inverse difference moment normalized_δ[[3](#_ENREF_3)] |

**Note:** δ represents the filter of Laplacian Gaussian filter, which could be 0, 1.0, 1.5, 2.0 and 2.5.

# Evaluation of the performance of rad-score based prognostic nomograms.

Based on the results of multivariate analysis, rad-score based nomogram and clinicopathological factors based nomogram were developed by using RMS package. Meanwhile, rad-score was added to TNM staging system and BCLC staging system to develop rad-score TNM nomogram and rad-score BCLC nomogram. C-index was used to evaluate the predictive accuracy (discrimination) of all nomograms and traditional staging systems. Then, the C-index value of rad-score based nomogram was compared with the C-index value of clinicopathological factors based nomograms to evaluate the incremental prognostic value of rad-score. The same comparison was performed for the rad-score TNM nomogram and TNM staging system, as well as for the rad-score BCLC nomogram and BCLC nomogram.

# Rad-score formula

***Rad-score****= 2.688195- 4.306105e-09× (Contrast_0) + 7.882485e-08× (Cluster Prominence_0) + 3.492191× (Information measure of correlation2_0) + 3.088437× (Inverse difference normalized (INN)-0)-2.511158× (Information measure of correlation2_2)-1.641851× (Energy_2.5*)

# References:

1. Haralick RM, Shanmugam K, Dinstein I (1973) Textural Features for Image Classification. IEEE TRANSACTIONS ON SYSTEMS, MAN, AND CYBERNETICS SMC-3:

2. Clausi DA (2002) An analysis of co-occurrence texture statistics as a function of grey level quantization. Can. J. Remote Sensing 28: 17

3. Tsatsoulis LSaC (1999) Texture Analysis of SAR Sea Ice Imagery Using Gray Level Co-Occurrence Matrices. IEEE Transactions on Geoscience and Remote Sensing 37: 15
