# Supplementary material for: Radiomics score: a potential prognostic imaging feature for postoperative survival of solitary HCC patients
Source: BMC Cancer. 2018 Nov 21;18:1148. doi: 10.1186/s12885-018-5024-z (PMC6249916; doi:10.1186/s12885-018-5024-z)
Supplement: Supplementary file 5 — Table S1. Association between rad-score and clinicopathological features in training cohort (DOCX 2509 kb) [file 12885_2018_5024_MOESM5_ESM.docx]

# Radiomics Score: a potential prognostic imaging feature for postoperative survival of solitary HCC patients

Bo-Hao Zheng^1,^ **^†^**, Long-Zi Liu^1,^ ^†^, Zhi-Zhi Zhang^5^, Jie-Yi Shi^1^, Liang-Qing Dong^1^, Ling-Yu Tian^1^, Zhen-bin Ding^1^, Yuan Ji ^3^, Sheng-Xiang Rao^4^, Jian Zhou^1^, Jia Fan^1,2^, Xiao-Ying Wang^1*^, and Qiang Gao^1, 6*^

**Authors' Affiliations:**

1 Department of Liver Surgery and Transplantation, Liver Cancer Institute, Zhongshan Hospital and Key Laboratory of Carcinogenesis and Cancer Invasion, Fudan University, Shanghai 200032, China

2 Institute of Biomedical Sciences, Fudan University, Shanghai 200032, China

3 Department of Pathology, Zhongshan Hospital, Fudan University, Shanghai 200032, China

4 Department of Radiology, Zhongshan Hospital, Fudan University, Shanghai 200032, China

5 Department of Hematology, Shanghai Jiao Tong University School of Medicine Affiliated Tongren Hospital.

6 State Key Laboratory of Genetic Engineering, Fudan University, Shanghai, PR China.

**^†^** These authors contributed equally to this work.

**Authors’ E-mails**

Bo-Hao Zheng: zbh930@sina.com; Long-Zi Liu: liulongzi@126.com; Zhi-Zhi Zhang: zzz3721@shtrhospital.com; Jie-Yi Shi：shi.jieyi@zs-hospital.sh.cn ; Liang-Qing Dong： lq.dong@outlook.com; Ling-Yu Tian: 17211210045@fudan.edu.cn ; Zhen-bin Ding：ding.zhenbin@zs-hospital.sh.cn; Yuan Ji: ji.yuan@zs-hospital.sh.cn; Sheng-Xiang Rao; rao.shengxiang@zs-hospital.sh.cn; Jian Zhou: zhou.jian@zs-hospital.sh.cn; Jia Fan: fan.jia@zs-hospital.sh.cn; Xiao-Ying Wang: wang.xiaoying1@zs-hospital.sh.cn; Qiang Gao: gaoqiang@fudan.edu.cn.

*** Corresponding Author Addresses:**

Dr. Qiang Gao, Department of Liver Surgery and Transplantation, Liver Cancer Institute, Zhongshan Hospital, Fudan University, 180 Fenglin Road, Shanghai 200032, China. Tel./fax: +86-021-64037181; E-mail: gaoqiang@fudan.edu.cn. or Dr. Xiao-Ying Wang, Department of Liver Surgery and Transplantation, Liver Cancer Institute, Zhongshan Hospital, Fudan University, 180 Fenglin Road, Shanghai 200032, China. Tel./fax: +86-021-64037181. E-mail: wang.xiaoying1@zs-hospital.sh.cn.

# Abstract

**Background**

Radiomics is an emerging field in oncological research. In this study, we aimed at developing a radiomics score (rad-score) to estimate postoperative recurrence and survival in patients with solitary hepatocellular carcinoma (HCC).

**Methods**

A total of 319 solitary HCC patients (training cohort: n=212; validation cohort: n=107) were enrolled. Radiomics features were extracted from the artery phase of preoperatively acquired computed tomography (CT) in all patients. A rad-score was generated by using the least absolute shrinkage and selection operator (lasso) logistic model. Kaplan-Meier and Cox's hazard regression analyses were used to evaluate the prognostic significance of the rad-score. Final nomograms predicting recurrence and survival of solitary HCC patients were established based on the rad-score and clinicopathological factors. C-index and calibration statistics were used to assess the performance of nomograms.

**Results**

Six potential radiomics features were selected out of 110 texture features to formulate the rad-score. Low rad-score positively correlated with aggressive tumor phenotypes, like larger tumor size and vascular invasion. Meanwhile, low rad-score was significantly associated with increased recurrence and reduced survival. In addition, multivariate analysis identified the rad-score as an independent prognostic factor (recurrence: Hazard ratio (HR): 2.472, 95% confident interval (CI): 1.339-4.564, p=0.004；survival: HR: 1.558, 95%CI: 1.022-2.375, p=0.039). Notably, the nomogram integrating rad-score had a better prognostic performance as compared with traditional staging systems. These results were further confirmed in the validation cohort.

**Conclusions**

The preoperative CT image based rad-score was an independent prognostic factor for the postoperative outcome of solitary HCC patients. This score may be complementary to the current staging system and help to stratify individualized treatments for solitary HCC patients.

**Keywords:** Hepatocellular Carcinoma; Prognosis; Multidetector Computed Tomography; Nomogram.

# Background

Hepatocellular carcinoma (HCC) is the fifth most common cancer and the second most common cause of cancer-related death worldwide (1). Current HCC staging systems, like Barcelona Clinic Liver Cancer (BCLC) staging system, indicate that hepatectomy is a potentially curative treatment for patients with early-stage HCC (2). However, postoperative recurrence is high, with 5-year rates reaching 70% (3, 4), suggesting that even in the same early-stage, patients have a diverse postoperative prognosis. Thus, the current staging systems still need improvement, for example, incorporating new risk factors for a better stratification of postoperative outcome. In fact, traditional staging systems mainly consist of pathological factors, like tumor size and vascular invasion, while tremendous information in preoperative computed tomography (CT) or magnetic resonance imaging (MRI) reflecting tissue intrinsic characters and heterogeneity (5-8) remains untapped. Recently, it has been reported that various imaging features were associated with pathological features and prognosis of the tumor and complementary to current staging systems, like rectal cancer and bladder cancer (9, 10). As such, new prognostic factors, like those derived from CT and MRI images, to identify patients with high risk of postoperative recurrence and death are urgently needed, which could help to select patients who are more likely to benefit from surgery.

Radiomics, an emerging and promising field, hypothesizes that medical images, including CT and MRI, could provide vivid and crucial information on tumor (11). By converting medical images into high-dimensional, mineable and quantitative features via high-throughput data extraction, radiomics method provides an unprecedented opportunity to improve decision-support in oncology at low cost and noninvasively. Currently, image examinations are routinely conducted for cancer patients, including HCC (12). Compared to developing new molecular biomarkers, radiomics method may not require additional physical or molecular tests and thus not increase the economic burden of patients. In addition, previous studies have demonstrated that quantitative radiomics features were associated with clinical prognosis and underlying genomic patterns across a range of cancer types, such as non-small cell lung cancer(13) and advanced nasopharyngeal carcinoma(14).

In HCC, contrast-enhanced computed tomography (CECT) has been widely used in the diagnosis due to its high specificity and sensitivity (12). Meanwhile, it had been reported that the characteristics of tumor CT images were associated with gene expression profiles, pathological features, and prognosis of HCC (11, 15-17). As far as we are concerned, image features could be divided into semantic features and agnostic features. Semantic features are commonly used in the radiology lexicon to describe regions of interest, including internal arteries, hypodense halos and so on, while agnostic features, like texture features, attempt to capture lesion heterogeneity though quantitative descriptors(11, 14, 18, 19). Previous studies preferred the clinical application of semantic features, as they were easy to acquire. Recently, growing concerns have been paid on the potential clinical application of agnostic features. For instance, Fu *et al.* investigated the prognostic significance of CT image texture features for advanced HCC patients receiving TACE (transarterial chemoembolization) (15). Another study has suggested that texture analysis was promising for HCC patient stratification for determining the suitability of liver resection vs. TACE (11). Furthermore, texture analysis has been reported for the potential for predicting postoperative hepatic insufficiency and assessing fibrosis (20). However, the prognostic significance of radiomics feature has been rarely investigated in HCC patients receiving hepatectomy.

In this study, we aimed at developing a rad-score derived from the preoperative CECT of solitary HCC patients, based on the assumption that such rad-score may help to identify patients who were at high risk of postoperative recurrence and death and improve clinical decision making for solitary HCC patients.

# Methods

**Patient selection and data collection**

Patient recruitment, as well as the inclusion and exclusion criteria, were presented in **Figure S1**. A total of 319 patients were enrolled and randomly divided into a training cohort (n=212) and validation cohort (n=107). The pathological diagnoses on all cases were reviewed and confirmed independently by two expert pathologists.

Baseline clinicopathological data were derived from medical records. Tumor differentiation was graded by the Edmondson grading system (21). Postoperative follow-up strategy and treatment strategy were according to a uniform guideline as we previously described (22, 23), and were listed in the **Supplementary Materials**. Ethical approval was obtained from the institutional review board of Zhongshan Hospital, and the informed consent requirement was waived. Time to recurrence (TTR) was defined as the interval between surgery and recurrence or the last observation for surviving patients without recurrence. Overall survival (OS) was defined as the interval between surgery and death or the last observation for surviving patients. The data were censored at the last follow-up for living patients.

**Quantitative imaging characteristics**

CT protocols and details of texture features are described in **Supplementary Materials**. Arterial phase CECT data were retrieved from the institution archive in dicom format and loaded to a personal laptop for further textural analysis. In this study, a total of 110 candidate radiomics features were generated from one image by using an in-house algorithm implemented in Matlab 2016a (MathWorks, Natick, MA, USA). For texture analysis, a region of interest (ROI) was delineated initially around the tumor outline of the largest cross-sectional area. Details of texture feature extraction are presented in **Figure S2**.

**Inter-observer and intra-observer reproducibility of radiomics feature extraction**

Sixty images were randomly chosen for evaluating the inter-observer reproducibility of the radiomics feature. All these images were reviewed by two radiologists with 10 (reader 1) and 5 years (reader 2) experience in abdominal CT interpretation. To assess the intra-observer reproducibility, reader 1 repeated the generation of texture features twice in a 1-week period followed the same procedure.

A two-way random, single measure (absolute agreement) intraclass correlation coefficients (ICC) was used to assess the differences between the features generated by reader 1 (first time) and those by reader 2, as well as between the twice-generated features by reader 1. An ICC value below 0.40 was considered poor reliability, fair for values between 0.41 and 0.59, good for values between 0.60 and 0.74, and excellent for values between 0.75 and 1.00. This is a descriptive statistic can be used when quantitative measurements are made on units that are organized into groups. It describes how strongly units in the same group resemble each other. Previously, it has been reported as a reliable method to evaluate the reproducibility of data (24, 25) and has been used in the radiomics research (26).

**Feature selection and rad-score building**

According to the Harrell’s guideline, the number of events should exceed the number of included covariates by at least 10 times in a multivariate analysis. Therefore, in our study, the least absolute shrinkage and selection operator (lasso) method combined with logistic regression(27), was used to select the most useful features in the training cohort. This method minimized a log partial likelihood subject to the sum of the absolute values of the parameters being bounded by a constant:

$\hat{\beta}=argmin \mathcal{l}\left( \beta\right)$, subject to $\sum\left| \beta_{j} \right|\leq s$

where, β ̂ is the obtained parameters, l(β) is the log partial likelihood of the logistic regression model, s﹥0 is a constant.

As a benefit of the absolute constraint, the lasso method shrinks coefficients and changes some coefficients to zero (28). Therefore, it can be used for the feature reduction and selection. In this study, the standardized constraint parameter s was set as 0.00013868 and lasso selected 6 nonzero coefficients ($\hat{\beta}$). Then, the logistic regression model was obtained with its outcome being the hazard rate at the fifth year after operation for individuals. The R software and “glmnet” package (R foundation for Statistical computing, Vienna, Austria, URL: http://www.R-project.org, 2016) were used for the lasso logistics regression model analysis.

**Statistical analysis**

Statistical analyses were performed using SPSS software (20.0; SPSS, Inc., Chicago, IL, USA) and R software (R Foundation for Statistical Computing, Vienna, Austria) with the “rms” package (R Foundation for Statistical Computing, Vienna, Austria). Continuous variables were compared using the Mann-Whitney U, while category variables were compared using Chi-squared or Fisher’s exact tests. X-tile (Yale University, New Haven, CT, USA) software was used to determine the optimal cut-off value of the rad-score, which is a graphical method that illustrates the presence of substantial tumor subpopulations and shows the robustness of the relationship between a biomarker and outcome by construction of a two-dimensional projection of every possible subpopulation (29, 30). Survival curves were depicted using Kaplan–Meier analysis (log-rank test). The Cox’s proportional hazards regression model was applied for univariate and multivariate analyses. “Rms” package was used to build nomogram models. The Harrell’s concordance index (C-index) and calibration curves were used to evaluate the nomogram models (31). Details of nomogram models were listed in the **Supplementary materials**. A two-sided value of *p* < 0.05 was considered statistically significant.

# Results

**Clinical characteristics of the patients**

No significant differences in clinicopathological features were observed between the two cohorts (**Table 1**). All patients were solitary HCC and received R0 resection. The mean follow-up time in training and validation cohorts was 52.7±21.6 months and 54.5±22.1 months, respectively. Overall survival rates at 1, 3, and 5 years after operation was 87%, 76% and 69% for training cohort and 88%, 75% and 72% for validation cohort, respectively.

**Results of inter-observer and intra-observer reproducibility of radiomics feature extraction**

Satisfactory inter- and intra-observer reproducibility of the texture feature extraction was achieved. The reproducibility of radiomics feature extraction was good between the two readers (ICC range: 0.71-0.95) or between reader 1’s first and second-extracted features (ICC range: 0.83-0.99). These results suggested that our radiomics feature values were highly reproducible.

**Development of the rad-score and its association with clinicopathological features.**

Six features were selected out of 110 texture features by using the lasso-logistic selection of the basis of 212 patients in the training cohort (**Figure S3**). The rad-score calculation formula consisting of these features was presented in **Supplementary Materials**. All the coefficients in the equation are from lasso-logistic regression. Determined by X-tile software, the optimal cut-off for rad-score was 4.32 (Rad-score range: Training cohort: 1.70-22.3; Validation cohort: 2.1-29.2). Accordingly, patients were divided into high (> 4.32) and low (≤ 4.32) groups.

Further investigation was performed to assess the association between the rad-score and clinicopathological features in the training cohort (**Table S1**). Patients with low rad-score were positively associated with high preoperative alpha-fetoprotein (AFP) level (p < 0.001), larger tumor size (*p* < 0.001), presence of vascular invasion (*p* = 0.009), advanced TNM stage (*p* = 0.015) and BCLC stage (*p* = 0.020), suggesting that low rad-score may indicate tumor aggressiveness.

**Low rad-score correlated with poor survival in solitary HCC patients**

In the training cohort, low rad-score were significantly associated with shorter TTR (median TTR [95% confident interval (CI)] for low [n=49] versus high rad-score [n=163]: 38 [28.2-47.1] versus 53 [48.0-58.4] months; *p* = 0.005, **Figure 1a**). In the validation cohort, no significance was observed in recurrence between the two groups with the *p* value of 0.054 (**Figure 1b**), suggesting that the rad-score was slightly over-fitted to the training cohort. As for OS, low rad-score significantly correlated with shorter postoperative survival in both training cohort (median OS [95% CI] for low [n=49] versus high rad-score [n=163]: 54.9[45.4-64.5] versus 70.5 [66.6-74.5] months; *p*=0.003, **Figure 1c**) and validation cohort (median OS [95%CI] for low [n=37] versus high [n=70]: 50.9[38.5-63.3] versus 82.2 [75.6-88.8] months; *p* = 0.003, **Figure 1d**).

Multivariate analyses suggested that rad-score was an independent prognostic factor of recurrence in the training cohort (Hazard ratio (HR): 2.472, 95%CI: 1.339-4.564, *p*=0.004, **Table 2**). As for OS, the rad-score (HR: 1.558, 95%CI: 1.022-2.375, *p*=0.039, **Table 3**) was also identified as an independent prognostic factor in the training cohort. Similar results were observed in the validation cohort (recurrence: HR: 1.890, 95%CI: 1.04-3.436, *p*=0.036, **Table 2**; survival: HR: 3.236, 95%CI: 1.416-7.407, *p*=0.005, **Table 3**).

All these results demonstrated that rad-score was an independent prognostic factor of postoperative recurrence and survival for solitary HCC patients. Patients with low rad-score have a higher recurrence rate and poorer survival.

**The performance of rad-score based prognostic nomograms**

Based on the results of multivariate analysis, rad-score based nomogram predicting postoperative recurrence (**Figure 2a**) of solitary HCC patients was established. In the nomogram model, each factor was ascribed a weighted point that implied a risk of recurrence or survival. For example, low rad-score was ascribed 20 points (on a scale of 0 - 100 points) in nomogram for postoperative survival. Each patient with a high total score had a worse prognosis, namely higher risk of recurrence or death. C-index was used to evaluate the predictive accuracy (discrimination) of the rad-score based nomograms, which was 0.639 (95% CI: 0.577-0.701, **Table 4**) for the nomogram of recurrence and 0.714 (95% CI: 0.635-0.793, **Table 4**) for the nomogram of survival in the training cohort. In the validation cohort, the C-index was 0.587(95% CI: 0.479-0.695, **Table 4**) for nomogram of recurrence, and the C-index was 0.71 (95% CI: 0.602-0.808, **Table 4**) for nomogram of survival. In addition, 50-sample bootstrapped calibration plots revealed the good predictive accuracy of the nomogram for the prediction of 3- (**Figure 2b and Figure 2c**) and 5- (**Figure 2d and Figure 2e**) year recurrence rate in the training and validation cohorts.

Similarly, rad-score based nomogram prediction postoperative survival of solitary HCC patients was developed (**Figure 3a**). Good predictive accuracy of 3- (**Figure 3b and Figure 3c**) and 5-(**Figure 3d and Figure 3e**) year survival rate was also observed in both training and validation cohorts.

Indeed, the Hosmer-Lemeshow test yielded no significant difference between the predictive calibration curve and the ideal curve for postoperative recurrence and survival prediction in both training and validation datasets. These results indicated that two nomograms could predict postoperative recurrence and survival effectively.

**Comparison between the rad-score based nomograms and traditional staging systems**

Previously, several traditional staging systems have been proposed for patients with HCC, including 7th edition of the American Joint Committee on Cancer (AJCC) TNM staging criteria, BCLC staging system(32), Japan Integrated Staging (JIS) (33) score and Hong Kong Liver Cancer (HKLC) staging score(34). In the training cohort, the C-index of these staging systems in predicting postoperative survival was 0.575 (95% CI: 0.515-0.635) for AJCC staging system, 0.574(95% CI: 0.511-0.637) for BCLC staging system, 0.601(95% CI: 0.533-0.669) for JIS staging system and 0.628(95% CI: 0.548-0.708) for HKLC staging system, respectively **(Table 4)**. When being compared to C-indices of our new nomogram including the rad-score, the C-indices of these staging systems were significantly lower in both training and validation cohorts. As for recurrence, the C-index of four staging systems was 0.552 (95% CI: 0.513-0.581) for AJCC TNM staging system, 0.547 (95% CI: 0.506-0.588) for BCLC staging system, 0.554 (95% CI: 0.508-0.600) for JIS staging system and 0.575 (95% CI: 0.529-0.631) for HKLC staging system, respectively, significantly lower than the C-index of our nomogram including the rad-score in both training and validation cohorts **(Table 4)**. All these results suggested that our rad-score based nomograms had a better discrimination performance than traditional staging system for solitary HCC patients.

**Assessment of incremental value of rad-score**

To investigate the incremental value of rad-score in individual postoperative recurrence and survival prediction, we compared the discrimination performance of clinicopathological nomograms and rad-score based nomograms. The clinicopathological nomograms were established based on independent clinicopathological risk factors, with the C-index of 0.633 (95% CI: 0.571-0.695) for recurrence and 0.554 (95% CI: 0.485-0.623) for postoperative survival in the training cohort. The discrimination performance of the nomogram improved when the rad-score was integrated (recurrence: C-index, 0.639, 95%CI: 0.577-0.701; survival: C-index, 0.714, 95%CI: 0.635-0.793), significantly higher than the discrimination performance of clinicopathological nomogram in the training cohort (**Table 4**). In the validation cohort, similar results were observed for postoperative survival. The C-index of clinicopathological nomogram was 0.642 (95%CI: 0.532-0.752), while the C-index (0.710, 95%CI: 0.602-0.818) improved after incorporating the rad-score into nomogram (**Table 5**). These results suggested that the rad-score was a good complementary to clinicopathological factors in individual postoperative recurrence and survival prediction.

The similar analysis was performed for traditional staging systems. An improvement in evaluating postoperative recurrence and survival was observed after combining the rad-score with the TNM staging system and BCLC staging system (**Table 4**). Hence, the rad-score is complementary to the TNM and BCLC staging system, demonstrating the valuable prognostic role of rad-score.

# Discussion

In this study, a multi-CT-texture feature based rad-score was proposed, which successfully stratified patients into groups with significant differences in TTR and OS, and may be complementary to traditional staging systems.

Radiomics, a promising field of oncological research, assume that image features could predict the prognosis of patients, as they are associated with tumor biological characteristics (11, 35). Previous studies have supported this hypothesis (17, 36). For instance, Banerjee *et al.* proposed an image features of venous invasion, consisting of three semantic features (internal arteries, hypodense halo, and tumor liver difference), were closely associated with early recurrence and poor survival for HCC (37). Similarly, the rad-score identified in our study was closely associated with pathological factors of HCC, like larger tumor size and vascular invasion and could be predictive of recurrence and survival.

Previously, several staging systems have been proposed for HCC patients, including TNM, BCLC, and HKLC (38). Our rad-score based nomograms yielded a better discriminative ability than these traditional staging systems for solitary HCC patients. In addition, our results suggested that the rad-score could complement the TNM and BCLC staging systems in prognostic stratification as the C-index value increased when the rad-score was added to them. This incremental ability indicated the clinical importance of our finding for solitary HCC patients.

In our study, lasso-logistic regression model was performed to select texture features to establish the rad-score, as features obtained from lasso were generally accurate and the regression coefficients of most features were shrunk toward zero during overfitting(39), making the model easier to interpret and allowing the identification of the most valuable features(40). Indeed, this method had been widely used in similar studies (14, 19).

Of note, the C-index values were relatively low for traditional staging systems, this phenomenon may be attributed to the study design. In our study, only solitary HCC patients were included. According to the traditional staging systems, these patients belong to the early or intermediate stages and are appropriate for surgery. Although they share the same or similar stage, a great deal of heterogeneity exists among them and they have a diverse postoperative prognosis. Thus, traditional staging systems could not actually predict recurrence and survival for these patients. In addition, the rad-score proposed also shared a relatively low C-index, but this couldn’t affect the clinical significance of rad-score, as it could stratify these patients into groups with different prognosis and improved the prognostic performance of traditional staging systems when being added into them for these patients.

The current study had several limitations. On one hand, the data in this study were derived from only one hepatobiliary center. On the other hand, only solitary HCC patients were included in this study, which may influence the generalization of the conclusion. In addition, this is a retrospective research. Therefore, further perspective multicenter analyses including HCC patients as various tumor stages were needed to validate the prognostic significance of this rad-score.

**Conclusions**

In summary, a rad-score derived from CT texture features was proposed in this study, which was an independent prognostic factor for tumor recurrence and survival of solitary HCC patients. In addition, this image score was complementary to the current staging systems of HCC patients. Finally, prognostic nomograms combining this score and clinicopathological features were proposed, which outperformed traditional staging systems and provided a convenient way to predict prognosis for solitary HCC patients, and may influence decision-making on the possible benefit of surgery.

# Abbreviations

**AFP,** alpha-fetoprotein

**AJCC,** American Joint Committee on Cancer

**ALB,** albumin

**ALT,** alanine aminotransferase

**AST,** aspartate aminotransferase

**BCLC,** Barcelona clinical liver cancer

**CECT,** contrast-enhanced computed tomography

**CI,** confidence interval

**C-index,** Harrell’s concordance index

**CT,** computed tomography

**DBIL,** direct bilirubin

**GGT,** γ-glutamyltransferase

**HCC,** hepatocellular carcinoma

**HKLC，**Hong Kong Liver Cancer

**HR,** hazard ratio

**ICC,** intraclass correlation coefficients

**JIS,** Japan Integrated Staging

**Lasso,** least absolute shrinkage and selection operator

**MRI,** magnetic resonance imaging

**OS,** overall survival

**Rad-score,** radiomics score

**ROI,** region of interest

**RVI,** radiogenomics venous invasion

**TAC**E, transarterial chemoembolization

**TBIL,** total bilirubin

**TNM,** tumor-node-metastasis

**TTR,** time to recurrence

# Declarations

**Acknowledgments**

Not applicable.

**Funding**

Supported by the National Natural Science Foundation of China (Nos. 81522036 and 81572292), Basic Research Project from Technology Commission of Shanghai Municipality (No. 17JC1402200), and National Program for Special Support of Eminent Professionals and Science. None of the funding sources were involved in design of the study, data collection and analysis, interpretation of results, writing of the manuscript, or in the decision to submit the manuscript for publication.

**Availability of data and materials**

The datasets used and analyzed during the current study are available from the corresponding author on reasonable request.

**Authors’ contributions**

X.Y.W and Q.G. conceived of the project and designed the research and drafted the manuscript. B.H.Z, L.Z.L, Z.Z.Z collected and organized the data, B.H.Z, Z.Z.Z, Y.L.T, J.Y.S. L.Q.D, Z.B.D, Y.J, S.X.R, J.Z, and J.F carried out the research, supervised the research, discussed its integrity and revised each part critically for publication, Q.G were responsible for quality control and managed the experimental design, reviewed the manuscript and provided funding support. All authors read and approved the final manuscript.

**Ethics approval and consent to participate**

The study with clinical data was approved by the Ethics Committee of the Zhongshan Hospital, Fudan University. We clarify that all clinical data in this study was collected in patients who had given written informed consent.

**Consent for publication**

Not applicable.

**Competing interests**

The authors declare that they have no competing interests.

**Publisher’s Note**

Springer Nature remains neutral with regard to jurisdictional claims in published maps and institutional affiliations.

**Open Access**

This article is distributed under the terms of the Creative Commons Attribution 4.0 International License (http://creativecommons.org/licenses/by/4.0/), which permits unrestricted use, distribution, and reproduction in any medium, provided you give appropriate credit to the original author(s) and the source, provide a link to the Creative Commons license, and indicate if changes were made. The Creative Commons Public Domain Dedication waiver (http://creativecommons.org/publicdomain/zero/1.0/) applies to the data made available in this article unless otherwise stated.

# References

(1) Siegel RL, Miller KD, and Jemal A Cancer Statistics, 2017*.* *CA Cancer J Clin* **2017**;67(1): p. 7-30 DOI: 10.3322/caac.21387.

(2) Forner A, Llovet JM, and Bruix J Hepatocellular carcinoma*.* *Lancet* **2012**;379(9822): p. 1245-55 DOI: 10.1016/S0140-6736(11)61347-0.

(3) Grazi GL, Ercolani G, Pierangeli F, Del Gaudio M, Cescon M, Cavallari A*, et al.* Improved results of liver resection for hepatocellular carcinoma on cirrhosis give the procedure added value*.* *Ann Surg* **2001**;234(1): p. 71-8.

(4) Zimmerman MA, Ghobrial RM, Tong MJ, Hiatt JR, Cameron AM, Hong J*, et al.* Recurrence of hepatocellular carcinoma following liver transplantation: a review of preoperative and postoperative prognostic indicators*.* *Arch Surg* **2008**;143(2): p. 182-8; discussion 188 DOI: 10.1001/archsurg.2007.39.

(5) Adhoute X, Penaranda G, Bronowicki JP, and Raoul JL Usefulness of the HKLC vs. the BCLC staging system in a European HCC cohort*.* *J Hepatol* **2015**;62(2): p. 492-3 DOI: 10.1016/j.jhep.2014.08.035.

(6) Liu PH, Hsu CY, Hsia CY, Lee YH, Su CW, Huang YH*, et al.* Prognosis of hepatocellular carcinoma: Assessment of eleven staging systems*.* *J Hepatol* **2016**;64(3): p. 601-8 DOI: 10.1016/j.jhep.2015.10.029.

(7) Farinati F, Vitale A, Spolverato G, Pawlik TM, Huo TL, Lee YH*, et al.* Development and Validation of a New Prognostic System for Patients with Hepatocellular Carcinoma*.* *PLoS Med* **2016**;13(4): p. e1002006 DOI: 10.1371/journal.pmed.1002006.

(8) Parikh ND, Scaglione S, Li Y, Powell C, Yerokun OA, Devlin P*, et al.* A Comparison of Staging Systems for Hepatocellular Carcinoma in a Multicenter US Cohort*.* *Clin Gastroenterol Hepatol* **2017**: DOI: 10.1016/j.cgh.2017.10.001.

(9) Liu Z, Zhang XY, Shi YJ, Wang L, Zhu HT, Tang Z*, et al.* Radiomics Analysis for Evaluation of Pathological Complete Response to Neoadjuvant Chemoradiotherapy in Locally Advanced Rectal Cancer*.* *Clin Cancer Res* **2017**;23(23): p. 7253-7262 DOI: 10.1158/1078-0432.CCR-17-1038.

(10) Wu S, Zheng J, Li Y, Yu H, Shi S, Xie W*, et al.* A Radiomics Nomogram for the Preoperative Prediction of Lymph Node Metastasis in Bladder Cancer*.* *Clin Cancer Res* **2017**;23(22): p. 6904-6911 DOI: 10.1158/1078-0432.CCR-17-1510.

(11) Gillies RJ, Kinahan PE, and Hricak H Radiomics: Images Are More than Pictures, They Are Data*.* *Radiology* **2016**;278(2): p. 563-77 DOI: 10.1148/radiol.2015151169.

(12) Chou R, Cuevas C, Fu R, Devine B, Wasson N, Ginsburg A*, et al.* Imaging Techniques for the Diagnosis of Hepatocellular Carcinoma: A Systematic Review and Meta-analysis*.* *Ann Intern Med* **2015**;162(10): p. 697-711 DOI: 10.7326/M14-2509.

(13) Liang C, Huang Y, He L, Chen X, Ma Z, Dong D*, et al.* The development and validation of a CT-based radiomics signature for the preoperative discrimination of stage I-II and stage III-IV colorectal cancer*.* *Oncotarget* **2016**;7(21): p. 31401-12 DOI: 10.18632/oncotarget.8919.

(14) Zhang B, Tian J, Dong D, Gu D, Dong Y, Zhang L*, et al.* Radiomics Features of Multiparametric MRI as Novel Prognostic Factors in Advanced Nasopharyngeal Carcinoma*.* *Clin Cancer Res* **2017**;23(15): p. 4259-4269 DOI: 10.1158/1078-0432.CCR-16-2910.

(15) Fu S, Chen S, Liang C, Liu Z, Zhu Y, Li Y*, et al.* Texture analysis of intermediate-advanced hepatocellular carcinoma: prognosis and patients' selection of transcatheter arterial chemoembolization and sorafenib*.* *Oncotarget* **2017**;8(23): p. 37855-37865 DOI: 10.18632/oncotarget.13675.

(16) Bakr S, Echegaray S, Shah R, Kamaya A, Louie J, Napel S*, et al.* Noninvasive radiomics signature based on quantitative analysis of computed tomography images as a surrogate for microvascular invasion in hepatocellular carcinoma: a pilot study*.* *J Med Imaging (Bellingham)* **2017**;4(4): p. 041303 DOI: 10.1117/1.JMI.4.4.041303.

(17) Segal E, Sirlin CB, Ooi C, Adler AS, Gollub J, Chen X*, et al.* Decoding global gene expression programs in liver cancer by noninvasive imaging*.* *Nature Biotechnology* **2007**;25(6): p. 675-680 DOI: 10.1038/nbt1306.

(18) Kickingereder P, Gotz M, Muschelli J, Wick A, Neuberger U, Shinohara RT*, et al.* Large-scale Radiomic Profiling of Recurrent Glioblastoma Identifies an Imaging Predictor for Stratifying Anti-Angiogenic Treatment Response*.* *Clin Cancer Res* **2016**;22(23): p. 5765-5771 DOI: 10.1158/1078-0432.CCR-16-0702.

(19) Nie K, Shi L, Chen Q, Hu X, Jabbour SK, Yue N*, et al.* Rectal Cancer: Assessment of Neoadjuvant Chemoradiation Outcome based on Radiomics of Multiparametric MRI*.* *Clin Cancer Res* **2016**;22(21): p. 5256-5264 DOI: 10.1158/1078-0432.CCR-15-2997.

(20) Daginawala N, Li B, Buch K, Yu H, Tischler B, Qureshi MM*, et al.* Using texture analyses of contrast enhanced CT to assess hepatic fibrosis*.* *Eur J Radiol* **2016**;85(3): p. 511-7 DOI: 10.1016/j.ejrad.2015.12.009.

(21) Gao Q, Wang XY, Qiu SJ, Yamato I, Sho M, Nakajima Y*, et al.* Overexpression of PD-L1 significantly associates with tumor aggressiveness and postoperative recurrence in human hepatocellular carcinoma*.* *Clin Cancer Res* **2009**;15(3): p. 971-9 DOI: 10.1158/1078-0432.CCR-08-1608.

(22) Xie DY, Ren ZG, Zhou J, Fan J, and Gao Q Critical appraisal of Chinese 2017 guideline on the management of hepatocellular carcinoma*.* *Hepatobiliary Surg Nutr* **2017**;6(6): p. 387-396 DOI: 10.21037/hbsn.2017.11.01.

(23) Zhou J, Sun H-C, Wang Z, Cong W-M, Wang J-H, Zeng M-S*, et al.* Guidelines for Diagnosis and Treatment of Primary Liver Cancer in China (2017 Edition)*.* *Liver Cancer* **2018**: p. 1-26 DOI: 10.1159/000488035.

(24) Bartko JJ The intraclass correlation coefficient as a measure of reliability*.* *Psychol Rep* **1966**;19(1): p. 3-11 DOI: 10.2466/pr0.1966.19.1.3.

(25) Shrout PE and Fleiss JL Intraclass correlations: uses in assessing rater reliability*.* *Psychol Bull* **1979**;86(2): p. 420-8.

(26) Aerts HJ, Velazquez ER, Leijenaar RT, Parmar C, Grossmann P, Carvalho S*, et al.* Decoding tumour phenotype by noninvasive imaging using a quantitative radiomics approach*.* *Nat Commun* **2014**;5: p. 4006 DOI: 10.1038/ncomms5006.

(27) Huang X and Pan W Linear regression and two-class classification with gene expression data*.* *Bioinformatics* **2003**;19(16): p. 2072-8.

(28) Tibshirani R The lasso method for variable selection in the Cox model*.* *Stat Med* **1997**;16(4): p. 385-95.

(29) Camp RL, Dolled-Filhart M, and Rimm DL X-tile: a new bio-informatics tool for biomarker assessment and outcome-based cut-point optimization*.* *Clin Cancer Res* **2004**;10(21): p. 7252-9 DOI: 10.1158/1078-0432.CCR-04-0713.

(30) Zheng BH, Yang LX, Sun QM, Fan HK, Duan M, Shi JY*, et al.* A New Preoperative Prognostic System Combining CRP and CA199 For Patients with Intrahepatic Cholangiocarcinoma*.* *Clin Transl Gastroenterol* **2017**;8(10): p. e118 DOI: 10.1038/ctg.2017.45.

(31) Iasonos A, Schrag D, Raj GV, and Panageas KS How to build and interpret a nomogram for cancer prognosis*.* *J Clin Oncol* **2008**;26(8): p. 1364-70 DOI: 10.1200/JCO.2007.12.9791.

(32) Llovet JM, Bru C, and Bruix J Prognosis of hepatocellular carcinoma: the BCLC staging classification*.* *Semin Liver Dis* **1999**;19(3): p. 329-38 DOI: 10.1055/s-2007-1007122.

(33) Kudo M, Chung H, and Osaki Y Prognostic staging system for hepatocellular carcinoma (CLIP score): its value and limitations, and a proposal for a new staging system, the Japan Integrated Staging Score (JIS score)*.* *J Gastroenterol* **2003**;38(3): p. 207-15 DOI: 10.1007/s005350300038.

(34) Yau T, Tang VY, Yao TJ, Fan ST, Lo CM, and Poon RT Development of Hong Kong Liver Cancer staging system with treatment stratification for patients with hepatocellular carcinoma*.* *Gastroenterology* **2014**;146(7): p. 1691-700 e3 DOI: 10.1053/j.gastro.2014.02.032.

(35) Schmitz J, Schwab J, Schwenck J, Chen Q, Quintanilla-Martinez L, Hahn M*, et al.* Decoding Intratumoral Heterogeneity of Breast Cancer by Multiparametric In Vivo Imaging: A Translational Study*.* *Cancer Research* **2016**;76(18): p. 5512-5522 DOI: 10.1158/0008-5472.can-15-0642.

(36) Kuo MD, Gollub J, Sirlin CB, Ooi C, and Chen X Radiogenomic analysis to identify imaging phenotypes associated with drug response gene expression programs in hepatocellular carcinoma*.* *J Vasc Interv Radiol* **2007**;18(7): p. 821-31 DOI: 10.1016/j.jvir.2007.04.031.

(37) Banerjee S, Wang DS, Kim HJ, Sirlin CB, Chan MG, Korn RL*, et al.* A computed tomography radiogenomic biomarker predicts microvascular invasion and clinical outcomes in hepatocellular carcinoma*.* *Hepatology* **2015**;62(3): p. 792-800 DOI: 10.1002/hep.27877.

(38) European Association For The Study Of The L, European Organisation For R, and Treatment Of C EASL-EORTC clinical practice guidelines: management of hepatocellular carcinoma*.* *J Hepatol* **2012**;56(4): p. 908-43 DOI: 10.1016/j.jhep.2011.12.001.

(39) Hepp T, Schmid M, Gefeller O, Waldmann E, and Mayr A Approaches to Regularized Regression - A Comparison between Gradient Boosting and the Lasso*.* *Methods Inf Med* **2016**;55(5): p. 422-430 DOI: 10.3414/ME16-01-0033.

(40) Ndhlovu ZM, Chibnik LB, Proudfoot J, Vine S, McMullen A, Cesa K*, et al.* High-dimensional immunomonitoring models of HIV-1-specific CD8 T-cell responses accurately identify subjects achieving spontaneous viral control*.* *Blood* **2013**;121(5): p. 801-11 DOI: 10.1182/blood-2012-06-436295.

# Figure Legends

**Figure 1 Prognostic significance of rad-score for solitary HCC patients.**

1. Training cohort: median TTR [95%CI] for low rad-score [n=49] versus high rad-score [n=163]: 38 [28.2-47.1] versus 53 [48.0-58.4] months; *p*=0.005
2. Validation cohort: *p*=0.054
3. Training cohort: median OS [95%CI] for low rad-score [n=49] versus high rad-score [n=163]: 54.9[45.4-64.5] versus 70.5 [66.6-74.5] months; *p*=0.003
4. Validation cohort: median OS [95%CI] for low rad-score [n=37] versus high rad-score [n=70]: 50.9[38.5-63.3] versus 82.2 [75.6-88.8] months; *p*=0.003

**Figure 2 Development of rad-score based nomograms and calibration curves of the rad-score based nomogram for recurrence in both training and validation cohorts.**

1. The prognostic nomogram for recurrence.
2. Calibration curves for 3 years TTR in the training cohort.
3. Calibration curves for 3 years TTR in the validation cohort.
4. Calibration curves for 5 years TTR in the training cohort.
5. Calibration curves for 5 years TTR in the validation cohort.

**Figure 3 Development of rad-score based nomograms and calibration curves of the rad-score based nomogram for OS in both training and validation cohorts.**

1. The prognostic nomogram for postoperative survival.
2. Calibration curves for 3 years OS in the training cohort.
3. Calibration curves for 3 years OS in the validation cohort.
4. Calibration curves for 5 years OS in the training cohort.
5. Calibration curves for 5 years OS in the validation cohort.

# Table Legends

**Table 1 Clinicopathological features of HCC patients in training and validation cohorts**

**Table 2** **Uni-and Multivariate analyses of predictors of postoperative recurrence in training and validation cohorts**

**Table 3** **Uni-and Multivariate analyses of predictors of postoperative survival in training and validation cohorts**

**Table 4 C-indices of rad-score based nomograms, clinicopathological nomograms and traditional staging systems**

# Additional files

**Additional files 1: Supplementary materials.** Details of methodology and results which were not shown in the manuscript.

**Additional files 2: Table S1 Association between rad-score and clinicopathological features in training cohort**

**Additional files 3: Figure S1 Flow chart of patient selection.**

**Additional files 4:** **Figure S2 The process of radiomics and the use of radioimics in decision support.**

(a) The acquisition of high-quality images.

(b) A region of interest (ROI) was identified by experienced radiologists.

(c) Texture features were extracted from ROI.

(d) These features were mined to develop prognostic models for clinical outcomes.

**Additional files 5:** **Figure S3 Texture feature selection using the least shrinkage and selection operator (lasso) binary logistic regression model.**

(a) Tuning parameter (λ) selection in the lasso model using 10-fold cross-validation via minimum criteria. The area under the receiver operating characteristic curve (AUC) was plotted versus log (λ). Dotted vertical lines were drawn at the optimal values by using the minimum criteria and the λ standard error of the minimum criteria (the 1-SE criteria). A value of 0.00013868, with log (λ) -3.858 was chosen (1-SE criteria) according to 10-fold cross-validation.

(b) Lasso coefficient profiles of the 110 texture features. A coefficient profile plot was produced against the log (λ) sequence. Vertical line was drawn at the value selected using 10-fold cross-validation, where optimal λ resulted in 6 nonzero coefficients.


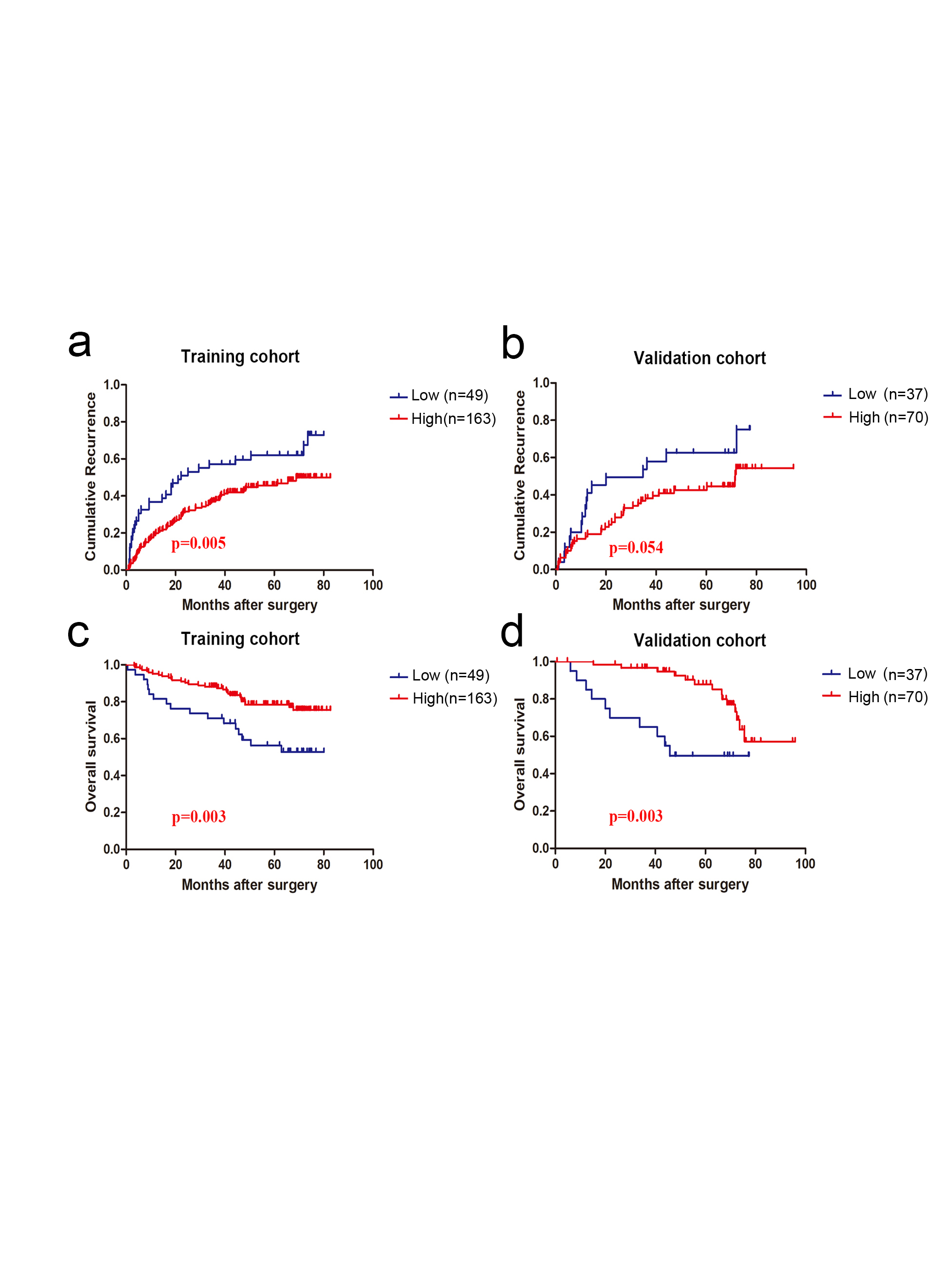


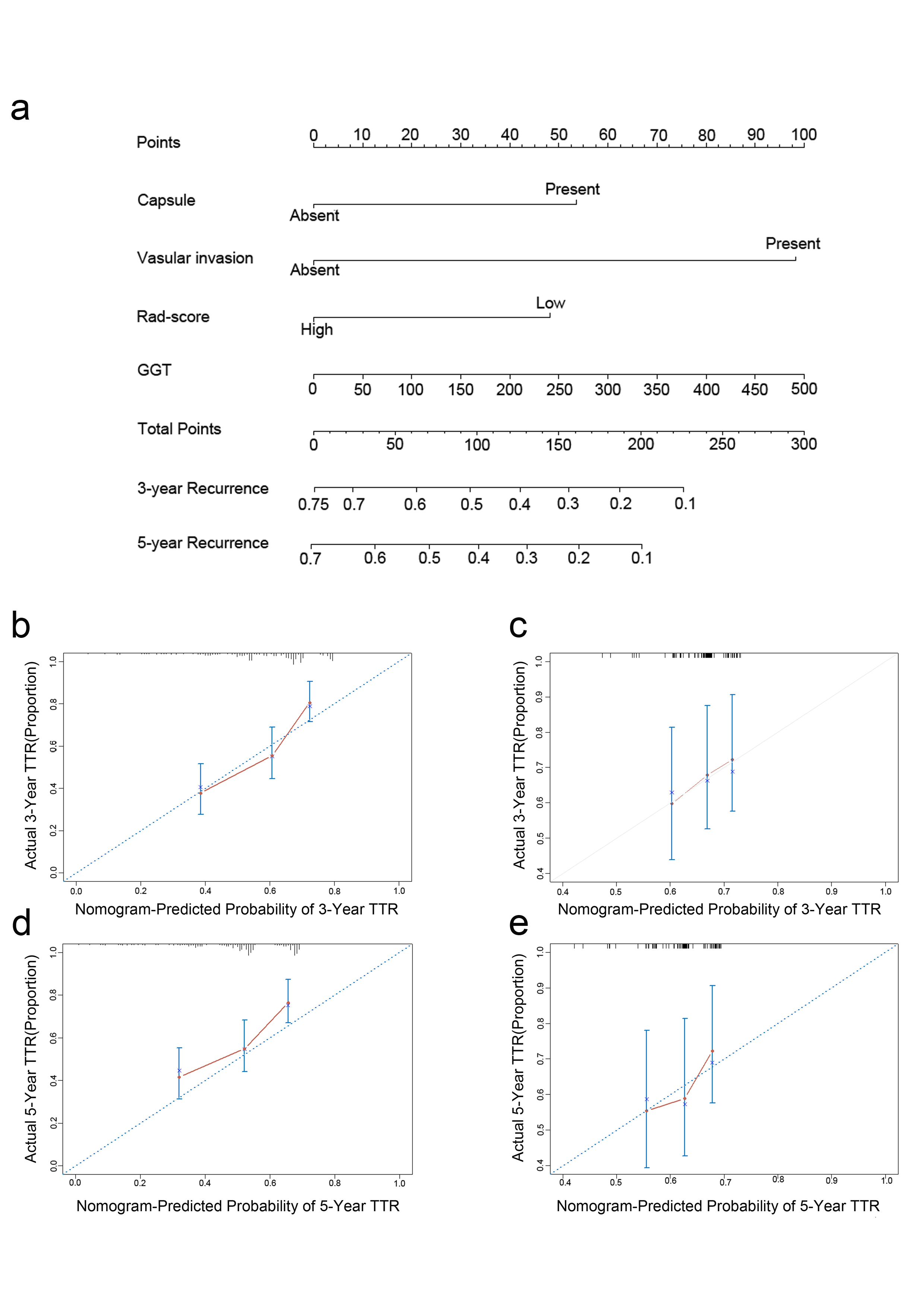


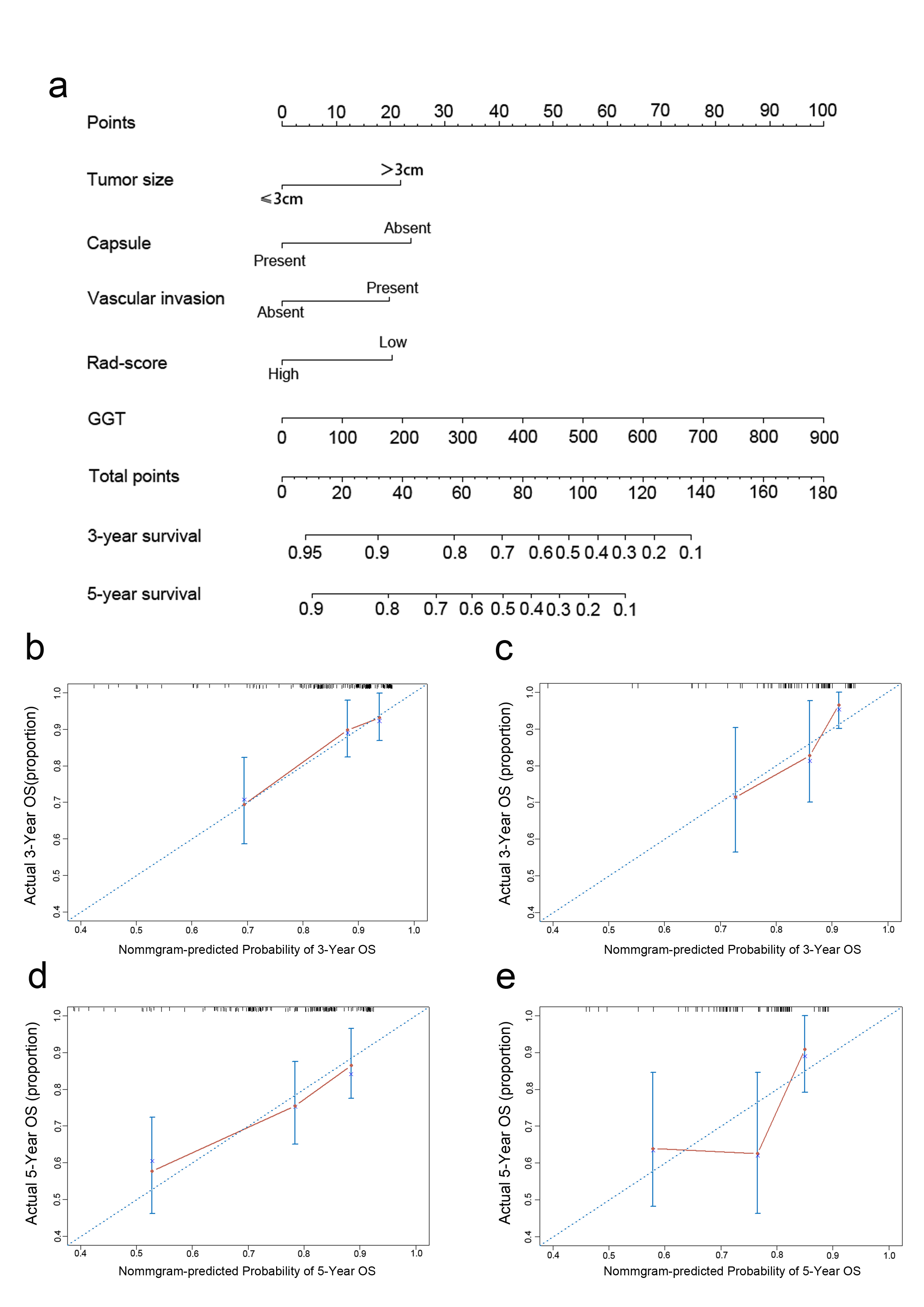


**Table 1 Clinicopathological features of HCC patients in training and validation cohorts**

| **Variable** | **Training cohort** | **Validation cohort** | ***p*** |
| --- | --- | --- | --- |
| **Median(range) age, y** | 55（24-85） | 55（13-83） | 0.93 |
| **Gender (male/female)** | 175/37 | 88/19 | 0.95 |
| **Tumor size(≤3,>3), cm** | 76/133 | 38/69 | 0.82 |
| **Vascular invasion(Present/Absent)** | 22/190 | 12/95 | 0.82 |
| **Microvascular invasion(Present/Absent)** | 68/144 | 29/78 | 0.36 |
| **Tumor differentiation**  **(III-IV/I-II)** | 16/193 | 6/101 | 0.46 |
| **Liver cirrhosis(Present/Absent)** | 166/46 | 87/20 | 0.53 |
| **HbsAg (Positive/Negative)** | 178/34 | 85/22 | 0.32 |
| **Tumor encapsulation (Present/ Absent)** | 112/110 | 59/48 | 0.70 |
| **Preoperative blood test** | | | |
| **DBIL(mean±SD), μmol/L** | 6.9±11.9 | 7.3±16.4 | 0.45 |
| **TBIL(mean±SD), μmol/L** | 14.8±14.5 | 15.04±19.2 | 0.94 |
| **ALT(mean±SD), U/L** | 40.1±31.3 | 46.1±69.2 | 0.91 |
| **AST(mean±SD), U/L** | 38.7±27.3 | 453.6±66.3 | 0.67 |
| **AFP(mean±SD), ng/ml** | 4460±13186 | 3800±12646 | 0.60 |
| **ALB(mean±SD), g/L** | 40.7±7.0 | 39.8±3.6 | 0.06 |
| **GGT(mean±SD), U/L** | 84.3±98.8 | 80.8±83.5 | 0.38 |

**Abbreviations:** ALB, albumin; ALT, alanine aminotransferase; AST, aspartate aminotransferase; DBIL, Direct Bilirubin; TBIL, total bilirubin; GGT, γ-glutamyltransferase; AFP, alpha-fetoprotein.

**Table 2** **Uni-and Multivariate analyses of predictors of postoperative recurrence in training and validation cohorts**

|  | | **Training cohort** | | | | | | **Validation cohort** | | |
| --- | --- | --- | --- | --- | --- | --- | --- | --- | --- | --- |
| **Variables** | | **Univariate** | | | **Multivariate** | | | **Multivariate** | | |
|  | | **HR** | **95%CI** | ***p*** | **HR** | **95%CI** | ***p*** | **HR** | **95%CI** | ***p*** |
| **Gender** | **Male** | 1.131 | 0.672-1.903 | 0.642 | \ | \ | \ | \ | \ | \ |
|  | **Female** |  |  |  |  |  |  |  |  |  |
| **Tumor size** | **≤3cm** | 1.831 | 1.190-2.817 | **0.006** | \ | \ | **0.193** | **\** | **\** | 0.167 |
|  | **>3cm** |  |  |  |  |  |  |  |  |  |
| **Vascular invasion** | **Present** | 2.364 | 1.341-4.166 | **0.003** | 2.602 | 1.408-4.807 | **0.002** | **\** | **\** | 0.141 |
|  | **Absent** |  |  |  |  |  |  |  |  |  |
| **Microvascular invasion** | **Present** | 1.285 | 0.858-1.926 | 0.223 | \ | \ | \ | \ | \ | \ |
|  | **Absent** |  |  |  |  |  |  |  |  |  |
| **Differentiation** | **III-IV** | 1.242 | 0.871-1.772 | 0.231 | \ | \ | \ | \ | \ | \ |
|  | **I-II** |  |  |  |  |  |  |  |  |  |
| **Liver Cirrhosis** | **YES** | 1.076 | 0.89-1.301 | 0.451 | \ | \ | \ | \ | \ | \ |
|  | **NO** |  |  |  |  |  |  |  |  |  |
| **HBsAg** | **Positive** | 1.292 | 0.747-2.233 | 0.359 | \ | \ | \ | \ | \ | \ |
|  | **Negative** |  |  |  |  |  |  |  |  |  |
| **Tumor encapsulation** | **Present** | 1.533 | 1.032-2.277 | **0.034** | 1.637 | 1.074-2.494 | **0.022** | **\** | **\** | 0.975 |
|  | **Absent** |  |  |  |  |  |  |  |  |  |
| **Rad-score** | **≤4.32** | 1.779 | 1.174-2.703 | **0.007** | 1.558 | 1.022-2.375 | **0.039** | **1.890** | **1.041-3.436** | **0.036** |
|  | **>4.32** |  |  |  |  |  |  |  |  |  |
| **DBIL, μmol/L** | | 1.002 | 0.986-1.019 | 0.797 | \ | \ | \ | \ | \ | \ |
| **TBIL, μmol/L** | | 1.003 | 0.99-1.015 | 0.691 | \ | \ | \ | \ | \ | \ |
| **ALT, U/L** | | 1.001 | 0.998-1.004 | 0.624 | \ | \ | \ | \ | \ | \ |
| **AST, U/L** | | 1.001 | 0.999-1.004 | 0.339 | \ | \ | \ | \ | \ | \ |
| **AFP, ng/ml** | | 1.000 | 1.000-1.000 | 0.494 | \ | \ | \ | \ | \ | \ |
| **ALB, g/L** | | 1.020 | 0.986-1.055 | 0.253 | \ | \ | \ | \ | \ | \ |
| **GGT, U/L** | | 1.002 | 1.001-1.004 | **0.002** | 1.002 | 1.000-1.003 | **0.017** | **\** | **\** | 0.426 |

**Abbreviations:** ALB, albumin; ALT, alanine aminotransferase; AST, aspartate aminotransferase; DBIL, Direct Bilirubin; TBIL, total bilirubin; GGT, γ-glutamyltransferase; AFP, alpha-fetoprotein.

**Table 3** **Uni-and Multivariate analyses of predictors of postoperative survival in training and validation cohorts**

|  | | **Training cohort** | | | | | | **Validation cohort** | | |
| --- | --- | --- | --- | --- | --- | --- | --- | --- | --- | --- |
|  | | **Univariate analysis** | | | **Multivariate analysis** | | | **Multivariate analysis** | | |
| **Variables** | | **HR** | **95%CI** | ***p*** | **HR** | **95%CI** | ***p*** | **HR** | **95%CI** | ***p*** |
| **Gender** | **Male** | 0.931 | 0.451-1.923 | 0.847 | \ | \ | \ |  |  |  |
|  | **Female** |  |  |  |  |  |  |  |  |  |
| **Tumor size(cm)** | **≤3** | 2.822 | 1.406-5.650 | **0.004** | 1.910 | 0.897-4.070 | **0.035** | **\** | **\** | 0.139 |
|  | **>3** |  |  |  |  |  |  |  |  |  |
| **Vascular invasion** | **Present** | 3.52 | 1.701-7.287 | **0.001** | 1.794 | 0.797-4.037 | **0.008** | **\** | **\** | 0.982 |
|  | **Absent** |  |  |  |  |  |  |  |  |  |
| **Micro-vascular invasion** | **Present** | 1.084 | 0.574-2.051 | 0.803 | \ | \ | \ | \ | \ | \ |
|  | **Absent** |  |  |  |  |  |  |  |  |  |
| **Differentiation** | **III-IV** | 1.515 | 0.886-2.592 | 0.129 | \ | \ | \ | \ | \ | \ |
|  | **I-II** |  |  |  |  |  |  |  |  |  |
| **Liver Cirrhosis** | **Yes** | 1.113 | 0.838-1.479 | 0.458 | \ | \ | \ | \ | \ | \ |
|  | **No** |  |  |  |  |  |  |  |  |  |
| **HBsAg** | **Present** | 1.557 | 0.662-3.664 | 0.31 | \ | \ | \ | \ | \ | \ |
|  | **Absent** |  |  |  |  |  |  |  |  |  |
| **Tumor encapsulation** | **Absent** | 2.105 | 1.155-3.846 | **0.015** | 2.326 | 1.272-4.255 | **0.006** | **\** | **\** | 0.394 |
|  | **Present** |  |  |  |  |  |  |  |  |  |
| **Rad-score** | **≤4.32** | 2.387 | 1.321-4.310 | **0.004** | 2.283 | 1.261-4.132 | **0.006** | 3.236 | 1.416-7.407 | **0.005** |
|  | **>4.32** |  |  |  |  |  |  |  |  |  |
| **DBIL, μmol/L** | | 1.015 | 0.998-1.031 | 0.084 | \ | \ | \ | \ | \ |  |
| **TBIL, μmol/L** | | 1.012 | 0.998-1.026 | 0.102 | \ | \ | \ | \ | \ | \ |
| **ALT, U/L** | | 0.998 | 0.991-1.005 | 0.57 | \ | \ | \ | \ | \ | \ |
| **AST, U/L** | | 1.000 | 0.995-1.005 | 0.946 | \ | \ | \ | \ | \ | \ |
| **AFP, ng/ml** | | 1.000 | 1.000-1.000 | 0.189 | \ | \ | \ | \ | \ | \ |
| **ALB, g/L** | | 1.020 | 0.964-1.079 | 0.495 | \ | \ | \ | \ | \ | \ |
| **GGT, U/L** | | 1.004 | 1.003-1.006 | **<0.0001** | 1.004 | 1.002-1.006 | **<0.0001** | **\** | **\** | 0.249 |

**Abbreviations:** ALB, albumin; ALT, alanine aminotransferase; AST, aspartate aminotransferase; DBIL, direct Bilirubin; TBIL, total bilirubin; GGT, γ-glutamyltransferase; AFP, alpha-fetoprotein.

**Table 4 C-indices of rad-score based nomograms, clinicopathological nomograms and traditional staging systems**

|  | **Training cohort** | | | | **Validation cohort** | | | |
| --- | --- | --- | --- | --- | --- | --- | --- | --- |
|  | **TTR** | | **OS** | | **TTR** | | **OS** | |
|  | **C-index** | **95%CI** | **C-index** | **95%CI** | **C-index** | **95%CI** | **C-index** | **95%CI** |
| **Rad-score** | 0.563 | 0.516-0.610 | 0.541 | 0.488-0.594 | 0.555 | 0.491-0.619 | 0.629 | 0.529-0.729 |
| **Rad-score based nomograms** | 0.639 | 0.577-0.701 | 0.714 | 0.635-0.793 | 0.587 | 0.479-0.695 | 0.710 | 0.602-0.818 |
| **Clinical-pathological nomograms** | 0.633 | 0.571-0.695 | 0.554 | 0.485-0.623 | 0.601 | 0.592-0.610 | 0.642 | 0.532-0.752 |
| **Rad-score-TNM nomogram** | 0.584 | 0.531-0.637 | 0.626 | 0.549-0.703 | 0.601 | 0.530-0.672 | 0.617 | 0.507-0.727 |
| **Rad-score-BCLC nomogram** | 0.580 | 0.526-0.634 | 0.627 | 0.549-0.705 | 0.601 | 0.523-0.679 | 0.622 | 0.509-0.735 |
| **Traditional staging system** | | | | | | | | |
| **TNM** | 0.552 | 0.513-0.581 | 0.575 | 0.515-0.635 | 0.553 | 0.499-0.607 | 0.521 | 0.453-0.589 |
| **BCLC** | 0.547 | 0.506-0.588 | 0.574 | 0.511-0.637 | 0.548 | 0.485-0.611 | 0.506 | 0.420-0.592 |
| **JIS** | 0.554 | 0.508-0.600 | 0.601 | 0.533-0.669 | 0.564 | 0.503-0.625 | 0.574 | 0.504-0.644 |
| **HKLC** | 0.575 | 0.529-0.631 | 0.628 | 0.620-0.636 | 0.582 | 0.507-0.657 | 0.596 | 0.487-0.701 |

**Abbreviation:** TNM, tumor–node–metastasis; BCLC, Barcelona Clinic Liver Cancer stage system; JIS, Japan Integrated Staging; HKLC, Hong Kong Liver Cancer staging score.
